# Multi-omics analysis-based clinical and functional significance of a novel prognostic and immunotherapeutic gene signature derived from amino acid metabolism pathways in lung adenocarcinoma

**DOI:** 10.3389/fimmu.2024.1361992

**Published:** 2024-12-13

**Authors:** Huihui Xiang, Rika Kasajima, Koichi Azuma, Tomoyuki Tagami, Asami Hagiwara, Yoshiro Nakahara, Haruhiro Saito, Yuka Igarashi, Feifei Wei, Tatsuma Ban, Mitsuyo Yoshihara, Yoshiyasu Nakamura, Shinya Sato, Shiro Koizume, Tomohiko Tamura, Tetsuro Sasada, Yohei Miyagi

**Affiliations:** ^1^ Molecular Pathology & Genetics Division, Kanagawa Cancer Center Research Institute, Yokohama, Japan; ^2^ Department of Pathology, Kanagawa Cancer Center, Yokohama, Japan; ^3^ Center for Cancer Genome Medicine, Kanagawa Cancer Center, Yokohama, Japan; ^4^ Department of Internal Medicine, Kurume University School of Medicine, Kurume, Japan; ^5^ Research Institute for Bioscience Products and Fine Chemicals, Ajinomoto Co., Inc., Kanagawa, Japan; ^6^ Department of Thoracic Oncology, Kanagawa Cancer Center, Yokohama, Kanagawa, Japan; ^7^ Department of Respiratory Medicine, Kitasato University School of Medicine, Sagamihara, Kanagawa, Japan; ^8^ Division of Cancer Immunotherapy, Kanagawa Cancer Center Research Institute, Yokohama, Japan; ^9^ Cancer Vaccine and Immunotherapy Center, Kanagawa Cancer Center, Yokohama, Japan; ^10^ Department of Immunology, Yokohama City University Graduate School of Medicine, Yokohama, Japan; ^11^ Morphological Analysis Laboratory, Kanagawa Cancer Center Research Institute, Yokohama, Japan

**Keywords:** prognostic gene signature, amino acid metabolism pathway, lung adenocarcinoma, multi-omics analysis, TP53 mutation, plasma-free α-aminobutyric acid

## Abstract

**Background:**

Studies have shown that tumor cell amino acid metabolism is closely associated with lung adenocarcinoma (LUAD) development and progression. However, the comprehensive multi-omics features and clinical impact of the expression of genes associated with amino acid metabolism in the LUAD tumor microenvironment (TME) are yet to be fully understood.

**Methods:**

LUAD patients from The Cancer Genome Atlas (TCGA) database were enrolled in the training cohort. Using least absolute shrinkage and selection operator Cox regression analysis, we developed PTAAMG-Sig, a signature based on the expression of tumor-specific amino acid metabolism genes associated with overall survival (OS) prognosis. We evaluated its predictive performance for OS and thoroughly explored the effects of the PTAAMG-Sig risk score on the TME. The risk score was validated in two Gene Expression Omnibus (GEO) cohorts and further investigated against an original cohort of chemotherapy combined with immune checkpoint inhibitors (ICIs). Somatic mutation, chemotherapy response, immunotherapy response, gene set variation, gene set enrichment, immune infiltration, and plasma-free amino acids (PFAAs) profile analyses were performed to identify the underlying multi-omics features.

**Results:**

TCGA datasets based PTAAMG-Sig model consisting of nine genes, *KYNU, PSPH, PPAT, MIF, GCLC, ACAD8, TYRP1, ALDH2*, and *HDC*, could effectively stratify the OS in LUAD patients. The two other GEO-independent datasets validated the robust predictive power of PTAAMG-Sig. Our differential analysis of somatic mutations in the high- and low-risk groups in TCGA cohort showed that the *TP53* mutation rate was significantly higher in the high-risk group and negatively correlated with OS. Prediction from transcriptome data raised the possibility that PTAAMG-Sig could predict the response to chemotherapy and ICIs therapy. Our immunotherapy cohort confirmed the predictive ability of PTAAMG-Sig in the clinical response to ICIs therapy, which correlated with the infiltration of immune cells (e.g., T lymphocytes and nature killer cells). Corresponding to the concentrations of PFAAs, we discovered that the high PTAAMG-Sig risk score patients showed a significantly lower concentration of plasma-free α-aminobutyric acid.

**Conclusion:**

In patients with LUAD, the PTAAMG-Sig effectively predicted OS, drug sensitivity, and immunotherapy outcomes. These findings are expected to provide new targets and strategies for personalized treatment of LUAD patients.

## 1 Introduction

Amino acid metabolism is crucial for tumor cell development and progression as a nitrogen and energy source in biosynthesis (1–3). Alterations in this metabolism, driven by intrinsic and extrinsic factors, impact both tumor and immune cells and shape cell fate, survival, proliferation, and metastasis (4–7). Tumor cells adapt to amino acid deficiencies in the tumor microenvironment (TME) by enhancing the uptake or synthesis of amino acids and regulating enzymes and transport proteins (8–10). Tumor cells compete to supply these resources to immune cells and inhibit their functions, aiding immune evasion (11). Additionally, catabolic processes play a critical role in the antitumor immune response (12–14). Conversely, immune cells influence tumor cell metabolism by releasing metabolites, including cytokines. For example, the release of interferon (IFN)-γ by activated T and natural killer (NK) cells can inhibit specific amino acid metabolic pathways in tumor cells, ultimately leading to tumor regression (15, 16).

The metabolic reprogramming of immune cells is closely related to the prognosis of patients with tumors and the efficacy of immunotherapy. Our previous clinical study on the metabolomics of approximately 200 cancer patients found significant differences in plasma-free amino acids (PFAAs) profile in patients with five types of cancer, including lung cancer, compared with healthy controls, even in those with asymptomatic early-stage diseases (17). The role of amino acid metabolism in immunotherapy is increasingly recognized, with studies demonstrating its importance in predicting survival in cancer patients treated with immune checkpoint inhibitors (ICIs) by circulating L-arginine, as well as predicting prognosis, immunogenicity, and efficacy of immunotherapy based on glutamine metabolism in lung adenocarcinoma (18, 19). We recently reported that a multivariate model with PFAAs and tryptophan metabolites in plasma might be helpful in stratifying patients who will benefit from PD-1 inhibitors (20). Accurate and direct evaluation of the predictive ability of amino acid metabolism on patient prognosis and exploring related mechanisms requires studying the expression of amino acid metabolism genes in tumor tissues. However, the relationship between the expression of genes in amino acid metabolism and prognosis of lung adenocarcinoma (LUAD) needs to be comprehensively investigated, and further studies are needed to confirm and explore this relationship in depth.

To pave the way for the development of personalized treatment strategies, we developed a novel prognostic signature, PTAAMG-Sig, based on the expression levels of genes involved in amino acid metabolism associated with overall survival (OS) in the LUAD, which could predict OS, drug sensitivity, and immunotherapy outcomes.

## 2 Materials and methods

### 2.1 Data collection from public databases

Data from 420 LUAD and 59 non-neoplastic lung tissues, including RNA sequencing, whole exome sequencing (WES), and patient clinical information, were retrieved from The Cancer Genome Atlas (TCGA) database (https://portal.gdc.cancer.gov/) in June 2020. The “maftools” R package was used to analyze WES data for somatic variants. Immunohistochemical staining (IHC) corresponding to TCGA-LUAD patients was acquired from the Human Protein Atlas (HPA) database (https://www.proteinatlas.org/) and the Clinical Proteomic Tumor Analysis Consortium (CPTAC) database (https://proteomics.cancer.gov/programs/cptac/). Microarray profiles of mRNA expression and clinical information of 353 patients were combined from the Gene Expression Omnibus (GEO) datasets GSE31210 and GSE50081. Another database containing data on 443 patients with LUAD was obtained from the GEO dataset GSE68465. We also obtained information from 24 non-small cell lung cancer (NSCLC) patients treated with PD-1 blockade combined with chemotherapy from the GEO GSE207422 dataset.

### 2.2 Original cohort

A clinical study on patients with advanced or recurrent stage III/IV NSCLC who were treated with cytotoxic chemotherapeutic reagents in combination with ICI therapy (pembrolizumab or atezolizumab) was performed from 2020 to 2022 at Kanagawa Cancer Center (KCC, Yokohama, Japan) and Kurume University Hospital (KU, Fukuoka, Japan). The treatment response of the patients was determined according to the Response Evaluation Criteria in Solid Tumors (RECIST) 1.1. From the cohort, 20 patients whose formalin-fixed paraffin-embedded (FFPE) tumor tissues were available for RNA sequencing, were involved in the present study. We named this cohort the KCC-ICI. For these patients, the concentrations of PFAAs in the peripheral venous blood before the start of treatment were examined. OS was defined as the period from the date of treatment to the date of death from any cause.

### 2.3 Construction of the amino acid metabolism-related genes (AAMGs) gene list

To comprehensively evaluate the role of amino acid metabolism, we identified genes related to amino acid metabolism. Briefly, all human genes related to 14 amino acid metabolic pathways were listed in the Kyoto Encyclopedia of Genes and Genomes (KEGG) (https://www.genome.jp/kegg/). The resultant 293 genes were designated as AAMGs. The entire list of genes by gene symbols is provided in **Supplementary Table 1**.

### 2.4 Identification of differentially expressed genes (DEGs) and subsequent pathway analysis

The “DESeq2” Bioconductor R package (v1.28.1) was used to normalize the process and identify the DEGs between the two groups from the RNA sequencing data (fold change > 2, *P*_adj_ < 0.05). Gene set enrichment analysis (GSEA) was performed based on the Gene Ontology (GO), KEGG, and Reactome Pathway (REACTOME) databases. The R packages “clusterProfiler” and “ReactomePA” were used for enrichment analysis and visualization of the results. Statistical significance was set at a normalized enrichment score |(NES)| > 1 and FDR < 0.05. Gene set variation analysis (GSVA) was performed to identify significantly correlated pathways using a reference gene set “c2.cp.kegg.v7.4. symbols.gmt” was downloaded from the GSEA website (https://www.gsea-msigdb.org/gsea/downloads.jsp), with an FDR < 0.05. The abundance of immune cell infiltration, stroma score, and immune score were determined by the ESTIMATE algorithm using RNA sequencing data.

### 2.5 Prediction of drug response

Drug response data for cytotoxic drugs in human cancer cell lines and the corresponding genomic markers were downloaded from the Genomics of Drug Sensitivity in Cancer (GDSC) website (http://www.cancerrxgene.org) as the GDSC v2 dataset. To predict the drug response profiles for patients involved in TCGA-LUAD cohort, analysis using the R package “oncoPredict” was applied to the GDSC v2 and RNA sequencing data of each LUAD tumor of the corresponding patient. The half-maximal inhibitory concentration (IC_50_) values of drugs were calculated for each patient, which were used to speculate how a drug inhibits certain biological or metabolic processes (21). Response to ICIs was predicted using the tumor immune dysfunction and exclusion (TIDE) algorithm based on the gene expression related to T cell dysfunction and exclusion, obtained from the TIDE website (http://tide.dfci.harvard.edu/) (22).

### 2.6 RNA sequencing of formalin-fixed paraffin-embedded (FFPE) tissues

For total RNA isolation from FFPE tissues of patients, we first marked the area of the tumor on the hematoxylin-eosin-stained section of each tissue block. Then, total RNA was extracted from the corresponding tumor area on the unstained serially sliced sections with macrodissection using RNeasy FFPE Kit (Qiagen, Hilden, Germany), according to the manufacturer’s instructions. The amount of RNA was measured using a NanoDrop1000 (Thermo Scientific, Wilmington, DE, USA) and RNA integrity was assessed using the RNA Nano 6000 Assay Kit of the Agilent Bioanalyzer 2100 system (Agilent Technologies, Santa Clara, CA). RNA samples with 30% or greater DV200 values were subjected to RNA sequencing. Construction of cDNA libraries followed by RNA sequencing was performed by Takara Bio Inc. (Shiga, Japan) as a contract analysis using a SMART-Seq Stranded mRNA Kit (Clontech, Palo Alto, CA, USA) and a NovaSeq sequencing system (Illumina, San Diego, CA, USA) according to the manufacturer’s instructions. After confirming the read quality using FastQC, the sequence data were aligned to the human genome GRCh37 using STAR-2.5.2a (https://github.com/alexdobin/STAR/releases/tag/2.5.2a), and the mapped read count of each sample was calculated using Python 2.7. Transcripts per million (TPM) were calculated for transcription quantification using Salmon-1.1.0 (https://combine-lab.github.io/salmon/) with GRCh38.v99 as the reference index. Salmon estimated TPM data were summarized to gene expression levels using the “tximport” package(v1.22.0) for correlation analysis. Mapping to the reference by STAR-2.5.2a and a quality check of reads for each sample were performed using the Genomon 2 analysis pipeline (https://github.com/Genomon-Project).

### 2.7 Analysis of PFAAs

Peripheral blood samples from the KCC-ICI cohort were collected in the morning in an overnight fasting state from the antecubital vein into tubes containing EDTA-2Na and immediately placed on ice. Plasma was separated via centrifugation at 3000 rpm for 15 min at 4°C and stored at −80°C until analysis. After thawing, plasma samples were deproteinized using acetonitrile at a final concentration of 50% before measuring amino acid concentrations using high-performance liquid chromatography–electrospray ionization mass spectrometry via precolumn derivatization, as described previously (20). Concentrations of the following 21 PFAAs were measured: alanine, arginine, asparagine, citrulline, α-aminobutyric acid (AABA), glutamate, glutamine, glycine, histidine, isoleucine, leucine, lysine, methionine, ornithine, phenylalanine, proline, serine, threonine, tryptophan, tyrosine, and valine. The amino acid concentration was determined by summing the concentrations of each of the 21 PFAAs.

### 2.8 Statistical analysis

For all statistical analyses, R version 4.1.0 was used, unless otherwise noted. Heatmaps were drawn by the “ComplexHeatmap” package with Spearman as a distance indicator; for the generation of Kaplan–Meier (KM) curves and calculation of Cox proportional hazard ratios, packages of the “survplot” and the “survminer” were used. Hazard ratios (HRs) and 95% confidence intervals (CIs) were calculated using the “Coxph” function. For the Concordance Index (C-index) and time-dependent AUC of receiver operating characteristic (ROC), the packages of the “dplyr” and the “survivalROC” were used. Univariate Cox regression and least absolute shrinkage and selection operator (LASSO) Cox regression analyses were performed by using the “glmnet” package in R. Wilcoxon rank sum test was used for differential significance test between the two groups. Categorical variables between groups were compared using Fisher’s exact chi-square test. A schematic overview of the study design is provided in **Supplementary Figure 1**.

## 3 Results

### 3.1 Landscape of amino acid metabolism pathway-related genes in tumor tissues of LUAD

We compared RNA gene expression between 420 TCGA-LUAD tumor samples and 59 non-neoplastic lung samples, and first identified 4543 significant DEGs (fold change > 2, P_adj_ < 0.05). We further identified 76 DEGs that appeared in the list of AAMGs and designated them as tumor-specific AAMGs (TAAMGs). Among the 76 TAAMGs, 61 genes were upregulated and 15 were downregulated in tumor tissues compared to those in non-neoplastic lung tissues (**Supplementary Figure 2A**). To gain insight into the function of TAAMGs, enrichment analysis with KEGG pathways was carried out; cysteine and methionine metabolism; arginine and proline metabolism; tyrosine metabolism; glycine, serine, and threonine metabolism; alanine, aspartate, and glutamate metabolism; biosynthesis of amino acids; and tryptophan metabolism were the most enriched terms (**Supplementary Figure 2B**).

### 3.2 Identification of prognosis-related TAAMGs and development of PTAAMG-Sig

**Figure 1:**
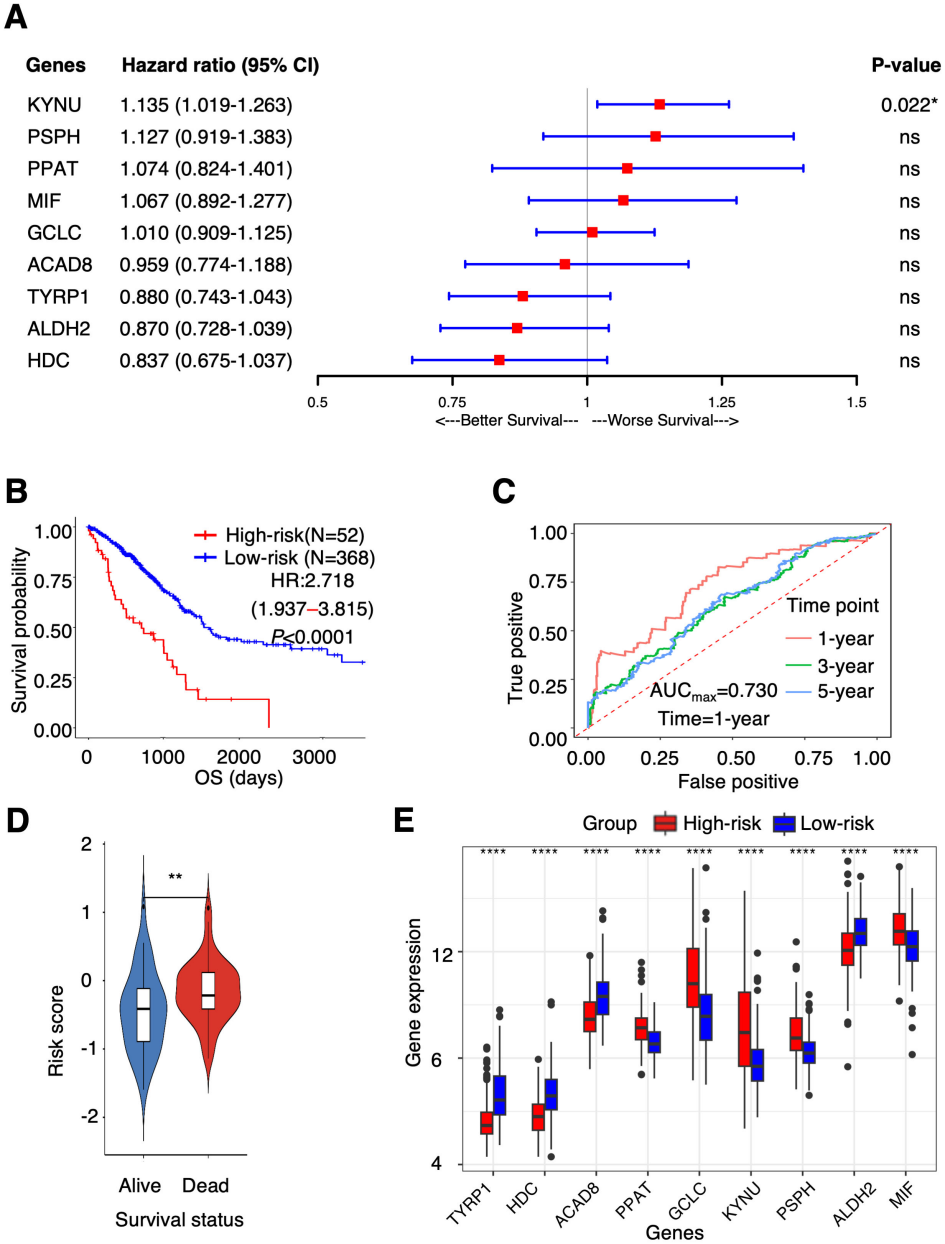
PTAAMG-Sig model was developed from the prognostic AAMGs. **(A)** Forest plot of the multivariate Cox regression analyses of the key genes in the PTAAMG-Sig model with OS. *P < 0.05; ns, not significant. **(B)** KM survival curve of the PTAAMG-Sig high- and low-risk patients in TCGA cohort. *P*-values by log-rank test. **(C)** The time-dependent ROC curves of the PTAAMG-Sig risk score at 1-, 3-, and 5 years of OS. **(D)** Violin plot comparing the distribution of risk scores between the alive and dead patients. Wilcoxon rank sum test, **P < 0.01. **(E)** Differential expressions of the key PTAAMGs in the PTAAMG-Sig high- and low-risk groups are depicted in a boxplot. Wilcoxon rank sum test, ****P < 0.0001. Genes are ordered based on *P*-values.

To identify TAAMGs related to patient prognosis, we first conducted a univariate Cox proportional hazards regression analysis using survival data of TCGA-LUAD patients and identified 16 candidate genes associated with OS with a *P* < 0.05 (these prognosis-related genes were designated as “PTAAMGs”) (**Supplementary Table 2**). Next, we established a prognostic signature composed of nine key PTAAMGs (*KYNU, PSPH, PPAT, MIF, GCLC, ACAD8, TYRP1, ALDH2*, and *HDC*) by selecting the mostly marked genes with the optimal value of tuning parameter (λ) by ten-time cross-validation using minimum criteria in LASSO Cox regression analysis. In addition, we performed a collinearity test to check the independence of the key PTAAMGs. The results showed that the multicollinearity assumption was not violated when the variance inflation factor (VIF) less than two. We named this novel prognostic signature model as “PTAAMG-Sig”, and the risk score of each patient was calculated using the expression values (EV) of optimized genes and their multivariate Cox regression correlation coefficients. The PTAAMG-Sig formulation was as follows: 0.0717*EV(PPAT) + 0.0649*EV(MIF) + 0.0095*EV(GCLC) + 0.1195*EV(PSPH) + 0.1262EV(KYNU) – 0.1394EV(ALDH2) – 0.0422EV(ACAD8) – 0.1782 EV(HDC) – 0.1274 EV(TYRP1). Multivariate Cox regression analysis of the key PTAAMGs comprising the signature showed that the expression of *KYNU, PSPH, PPAT, MIF*, and *GCLC* contributed to poorer OS with HRs > 1, whereas those of *ACAD8, TYRP2, ALDH2*, and *HDC* were associated with better OS with HRs < 1. Only KYNU had a *P*-value less than 0.05 (*P* = 0.022), indicating that it was a risk factor of this prognosis-predicting model (**Figure 1A**). The KM curves for each of the nine key PTAAMG expression levels revealed a significant association with OS (**Supplementary Figure 3**). When PTAAMG-Sig was applied to dichotomize TCGA-LUAD dataset with the risk score, 52 patients were classified into the high-risk group, with a value of −0.366 as the risk score cutoff. The high-risk group had significantly shorter OS than the low-risk group. Thus, PTAAMG-Sig efficiently stratified patient OS (C-index = 0.641, HR = 2.718, 95% CI 1.937–3.815, Log-rank *P* < 0.0001) (**Figure 1B**). We evaluated the signature using the ROC curve and calculated the area under the curve (AUC) at different time points (1, 3, and 5 years). The maximum AUC (AUC_max_) of PTAAMG-Sig was 0.730 at the 1-year OS time point (**Figure 1C**). We found that patients who died showed a significantly higher risk score than those who survived (Wilcoxon rank sum test, *P* < 0.01), indicating the effect of the risk score by PTAAMG-Sig on survival status (**Figure 1D**). These data suggest that the model efficiently stratifies the OS of LUAD patients. The expression patterns of the key PTAAMGs genes used in PTAAMG-Sig showed that the expression levels of *KYNU, PSPH, PPAT, MIF*, and *GCLC* were significantly higher in the high-risk group, whereas those of *ACAD8, TYRP2, ALDH2*, and *HDC* were significantly higher in the low-risk group (Wilcoxon rank sum test, *P* < 0.0001) (**Figure 1E**).

Based on the analysis of the HPA and CPTAC databases, we found that the protein expression levels of PPAT, KYNU, PSPH, ALDH2, and MIF evaluated by IHC in LUAD tumor tissues were consistent with the mRNA expression levels of each molecule; that is, high expression of PPAT, KYNU, PSPH, and MIF, as well as low ALDH2 expression, was observed in tumor tissues compared with those in the adjacent non-neoplastic lung tissues (**Supplementary Figures 4A, B**). In addition, we checked the mutation landscape of the nine genes in TCGA-LUAD tumor tissues. In total, 9.6% of patients had nonsynonymous gene mutations, such as missense, nonsense, frameshift insertion, or splice site mutations (**Supplementary Figure 5A**). *KYNU* had the highest mutation rate (3%), followed by *HDC* (2%). *PPAT* and *MIF* showed no mutations in TCGA-LUAD samples. The results of the multi-omics analyses revealed that the nine key genes exhibited consistent expression patterns at the protein and mRNA levels and had low mutation rates.

### 3.3 Validation of the predictive capability of PTAAMG-Sig in other independent datasets

To validate the predictive capability of PTAAMG-Sig, we investigated two independent lung cancer cohorts 2 public GEO cohorts of 353 patients (GSE31210 and GSE50081) and another GEO cohort of 443 patients (GSE68465). The combined GSE31210 and GSE50081 cohorts did not enroll patients at stage III and more advanced stages, whereas the GSE68465 cohort included stage III and stage IV patients with patients at the earlier stages. Detailed clinical information for TCGA and GEO cohorts is presented in **Table 1**.

**Table 1:** Clinical information of patients providing comprehensive gene expression profiles of tumors.

Patient characteristics	Training series	Validation series		ICI therapy series	
	TCGA	GSE31210, GSE50081	GSE68465	KCC_ICI	GSE207422
**Platform**	Illumina HiSeq	HG-U133_ Plus_2	HG-U133A	Illumina HiSeq	Illumina NovaSeq
**Patients, N**	420	353	443	20	24
**Age (years)**	65.4 (9.93)	62.9 (9.39)	64.4 (10.1)	64.7 (8.27)	60.9 (10.7)
Sex:					
**Female**	221 (52.60%)	183(51.80%)	220 (49.7%)	4 (20.00%)	5 (20.80%)
**Male**	199 (47.40%)	170(48.20%)	223 (50.3%)	16(80.00)	19 (79.20%)
Status:					
**Alive**	266 (63.30%)	267(75.60%)	207 (46.7%)	17 (85.0%)	–
**Dead**	154 (36.70%)	86 (24.40%)	236 (53.3%)	3 (15.0%)	–
Stage:					
**I**	226 (53.80%)	260(73.60%)	150(33.86%)	0 (0%)	2 (8.33%)
**II**	102 (24.30%)	93 (26.40%)	251(56.66%)	0 (0%)	8 (33.33%)
**III**	70 (16.70%)	0 (0%)	28 (6.32%)	2 (10.0%)	14 (58.33%)
**IV**	22 (5.20%)	0 (0%)	12 (2.71%)	15 (75.0%)	0 (0%)
**Recurrence**	0 (0%)	0 (0%)	0 (0%)	3 (15.0%)	0 (0%)
**NE**	0 (0%)	0 (0%)	2 (0.45%)	0 (0%)	0 (0%)

The clinical characteristics of patients for comprehensive transcriptome analysis are demonstrated.

Continuous variables are described with mean and standard deviation. Categorical variables are summarized as sample numbers and percentages.

Platform: Gene expression files of TCGA and KCC-ICI cohorts were obtained via RNA sequencing, and those of GEO cohorts were obtained using a microarray.

**Figure 2:**
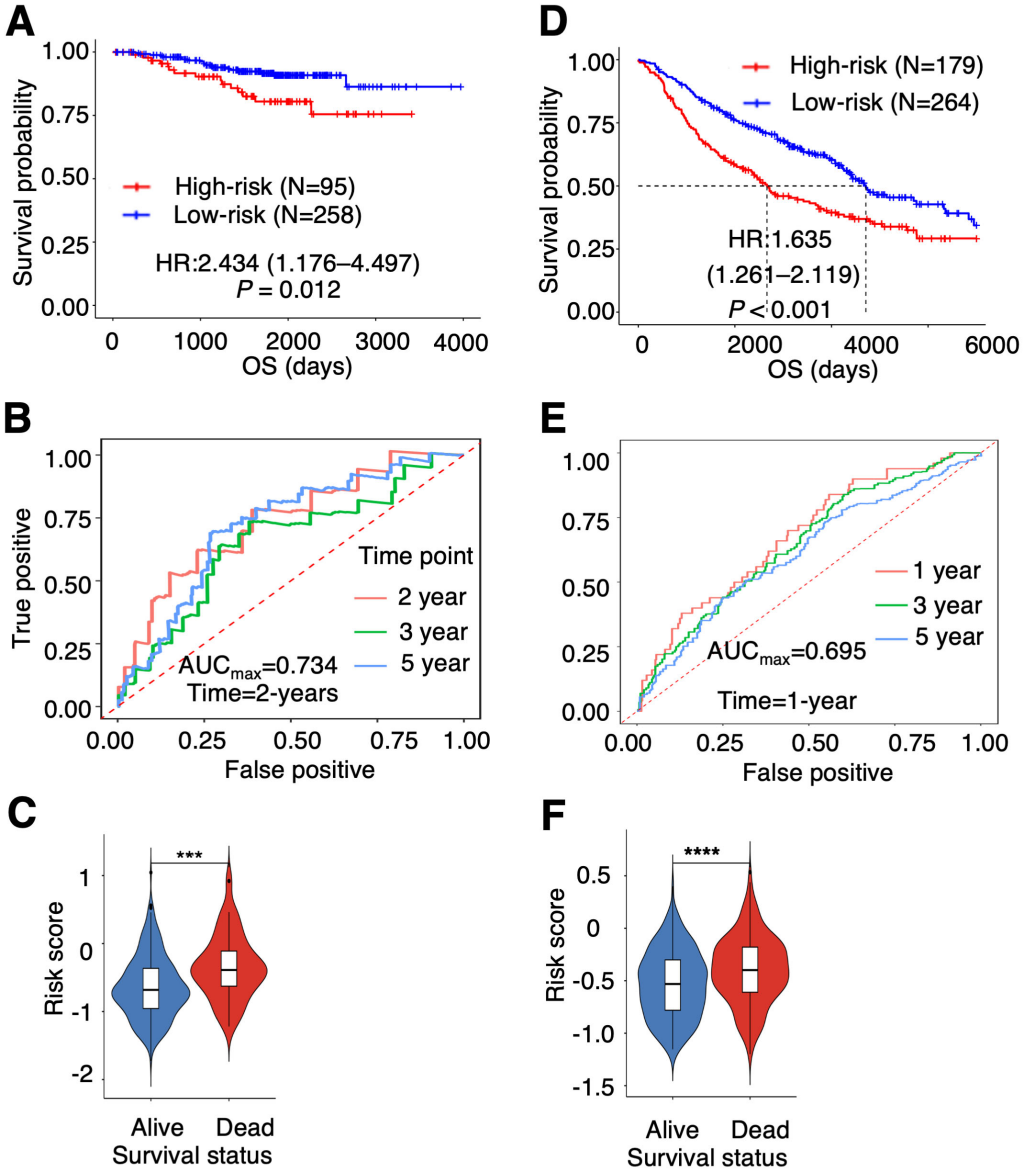
Validation of the prognostic PTAAMG-Sig model in independent cohorts. **(A)** KM survival curve of the PTAAMG-Sig high- and low-risk groups in the GEO cohort of 353 patients with LUAD combining the datasets GSE31210 and GSE50081. *P*-values by log-rank test. **(B)** The time-dependent ROC curves of the PTAAMG-Sig risk score at 2, 3, and 5 years of OS in the GEO cohort. **(C)** Violin plot comparing the distribution of risk scores between the alive and dead patients in the GEO cohort. Wilcoxon rank sum test, ***P < 0.001. **(D)** KM survival curves of the PTAAMG-Sig high-risk and low-risk groups in the GSE68465. *P*-values by log-rank test. **(E)** The time-dependent ROC curves of the PTAAMG-Sig risk score at 1, 3, and 5 years of OS in the GSE68465 cohort. **(F)** Violin plot comparing the distribution of risk scores between alive and dead patients in the GSE68465. Wilcoxon rank sum test, ****P < 0.0001.

After removing the batch effect of the gene expression files, we performed Cox regression survival analysis in each cohort. In the GEO cohorts, PTAAMG-Sig stratified patients into high-risk (N = 95) and low-risk (N = 258) groups with a significant correlation to OS (HR = 2.434, 95% CI 1.176–4.497, Log-rank *P* = 0.012) (**Figure 2A**). Time-dependent ROC analysis showed a maximum predictive accuracy of 0.734 at the 2-year time point (**Figure 2B**). Compared with the alive patients, the dead patients showed significantly higher risk scores (Wilcoxon rank sum test, *P* < 0.001) (**Figure 2C**). Further validation of the signature with another GEO cohort, GSE68465, was performed because the cohort included stages I–IV, and the composition of patient stages was closer to that of TCGA patients. PTAAMG-Sig effectively stratified patients in the GSE68465 into high-risk (N = 179) and low-risk (N = 264) groups and revealed a significant association with OS (HR = 1.635, 95% CI 1.261–2.119, log-rank *P* < 0.001) (**Figure 2D**). Time-dependent ROC analysis showed a peak predictive accuracy of 0.695 at one year (**Figure 2E**). Patients who died had significantly higher risk scores than those who survived (Wilcoxon rank sum test, *P* < 0.0001) (**Figure 2F**).

### 3.4 Evaluation of the PTAAMG-Sig in other independent cohorts of ICI including therapy

**Figure 3:**
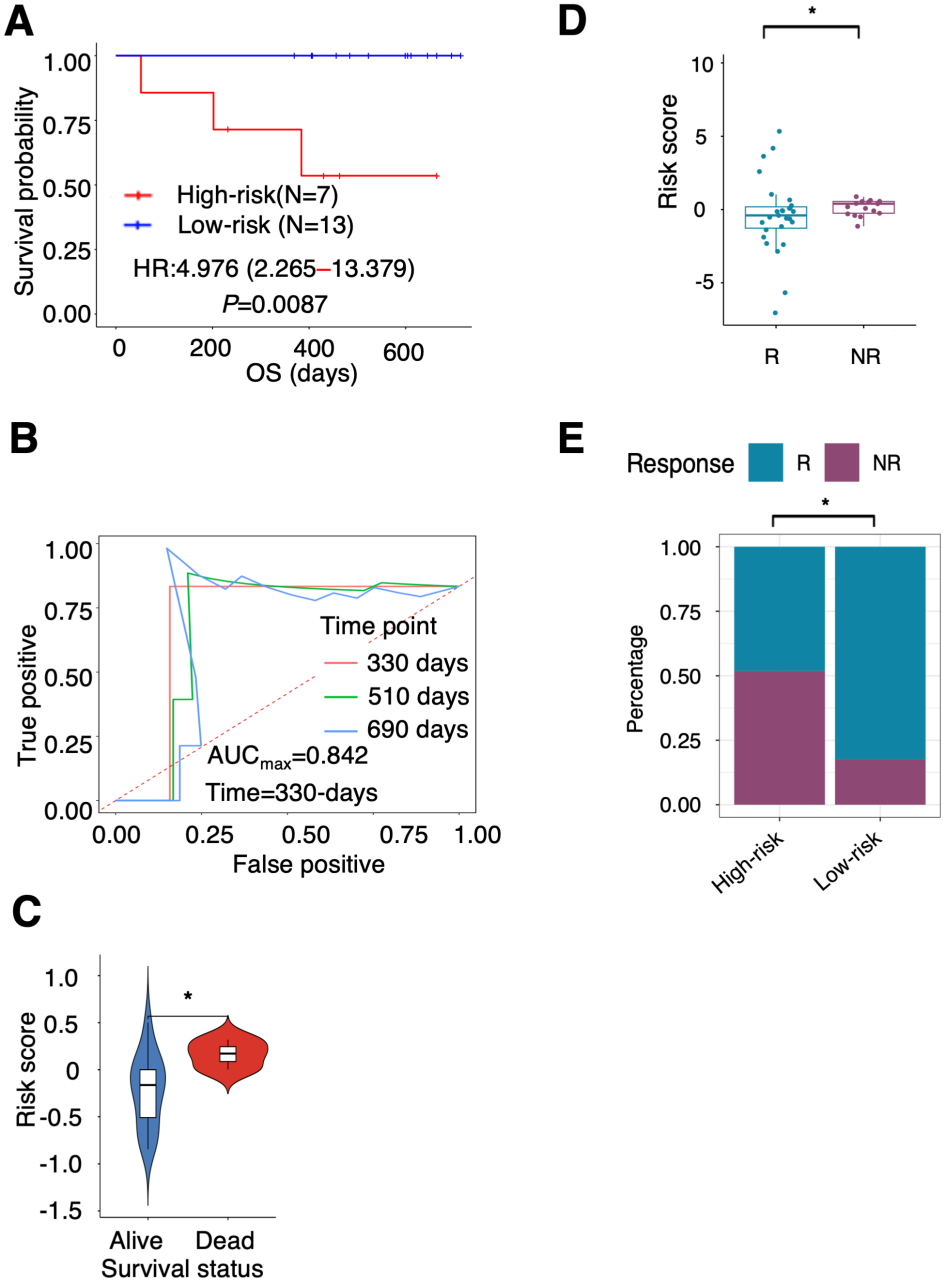
Ability of PTAAMG-Sig model to predict response to immunotherapy. **(A)** KM survival curves of the PTAAMG-Sig high-risk and low-risk groups in the KCC-ICI cohort. *P*-values by log-rank test. **(B)** The time-dependent ROC curves of the PTAAMG-Sig risk score at 330, 510, and 690 days of OS in the KCC-ICI cohort. **(C)** Violin plot comparing the distribution of risk scores between alive and dead patients in the KCC-ICI cohort. Wilcoxon rank sum test, *P < 0.05. **(D)** Boxplot showing the distribution of the PTAAMG-Sig risk scores in patients with different immunotherapeutic responses in a combination cohort of KCC-ICI and GSE207422. R: Responders; NR: Non-responders. Wilcoxon rank sum test, *P < 0.05. **(E)** The proportion distribution of patients with immunotherapeutic responses in the PTAAMG-Sig high- and low-risk groups in a combination cohort of KCC-ICI and GSE207422. R, Responders; NR, Non-responders. Chi-squared test, *P < 0.05.

TCGA cohort, and the GEO cohorts used for the validation of the PTAAMG-Sig patients were those before introduction of ICI to clinics. Therefore, we interrogated the efficacy of the PTAAMG-Sig in our original cohort enrolled 20 patients with NSCLC at advanced stages III/IV or with recurrence who received combined cytotoxic reagents and ICI therapy (KCC-ICI cohort). Univariate Cox regression analysis revealed no significant association between OS and sex, age, or stage (*P* > 0.05) (**Supplementary Table 3**). The patients were divided into high-risk (N = 7) and low-risk (N = 13) groups based on PTAAMG-Sig, and a good stratification ability of OS was confirmed in this cohort (HR = 4.976, 95% CI 2.265–13.379, Log-rank *P* = 0.0087) (**Figure 3A**). The maximum predictive accuracy of this signature was 0.842 at the 330-day time point (**Figure 3B**). Surviving patients showed substantially lower risk scores than those who died owing to PTAAMG-Sig (Wilcoxon rank sum test, *P* < 0.05) (**Figure 3C**).

To partly strengthen the results obtained from this small cohort, we analyzed the GEO dataset GSE207422, which enrolled 24 patients with NSCLC who received neoadjuvant PD-1 blockade in combination with chemotherapy followed by surgical resection of the tumors (**Table 1**). This dataset did not include information on OS but considered information on pathological responses to treatment after resection of the tumor. Nine patients were categorized as responders (with a major pathologic response defined as less than or equal to 10% viable tumor cells identified), and 15 were categorized as non-responders (non-major pathologic response). The responders had significantly lower PTAAMG-Sig risk scores than the non-responders (Wilcoxon rank sum test, *P* < 0.05) (**Supplementary Figure 6A**). Of patients with the PTAAMG-Sig high-risk scores, 28.6% were responders to ICI treatment, whereas all patients with low-risk scores were responders, and the difference was significant (chi-square test, *P* < 0.01) (**Supplementary Figure 6B**). We further combined the KCC_ICI and GSE207422 datasets and analyzed them as a cohort of 44 patients. The responders had significantly lower PTAAMG-Sig risk scores than the non-responders (Wilcoxon rank sum test, *P* < 0.05) (**Figure 3D**). Of the patients with PTAAMG-Sig high-risk scores, 51.9% were non-responders to ICI treatment, whereas 17.6% of the patients with low-risk scores were non-responders, and the difference was significant (chi-square test, *P* < 0.01) (**Figure 3E**).

Further evaluation of PTAAMG-Sig was performed in subgroups stratified by PD-L1 expression level in tumors. High PD-L1 expression with a tumor proportion score (TPS) ≥ 50% (n = 8) and PD-L1 positive expression with a TPS ≥ 1% (n = 13) were not significantly associated with OS (**Supplementary Figure 7A, B**). Combined with PTAAMA-Sig, we found that the high-risk patients in the low PD-L1 group had the shortest OS (log-rank *P* < 0.05) (**Supplementary Figure 7C**).

### 3.5 Somatic gene mutation profiles of the PTAAMG-Sig risk groups

**Figure 4:**
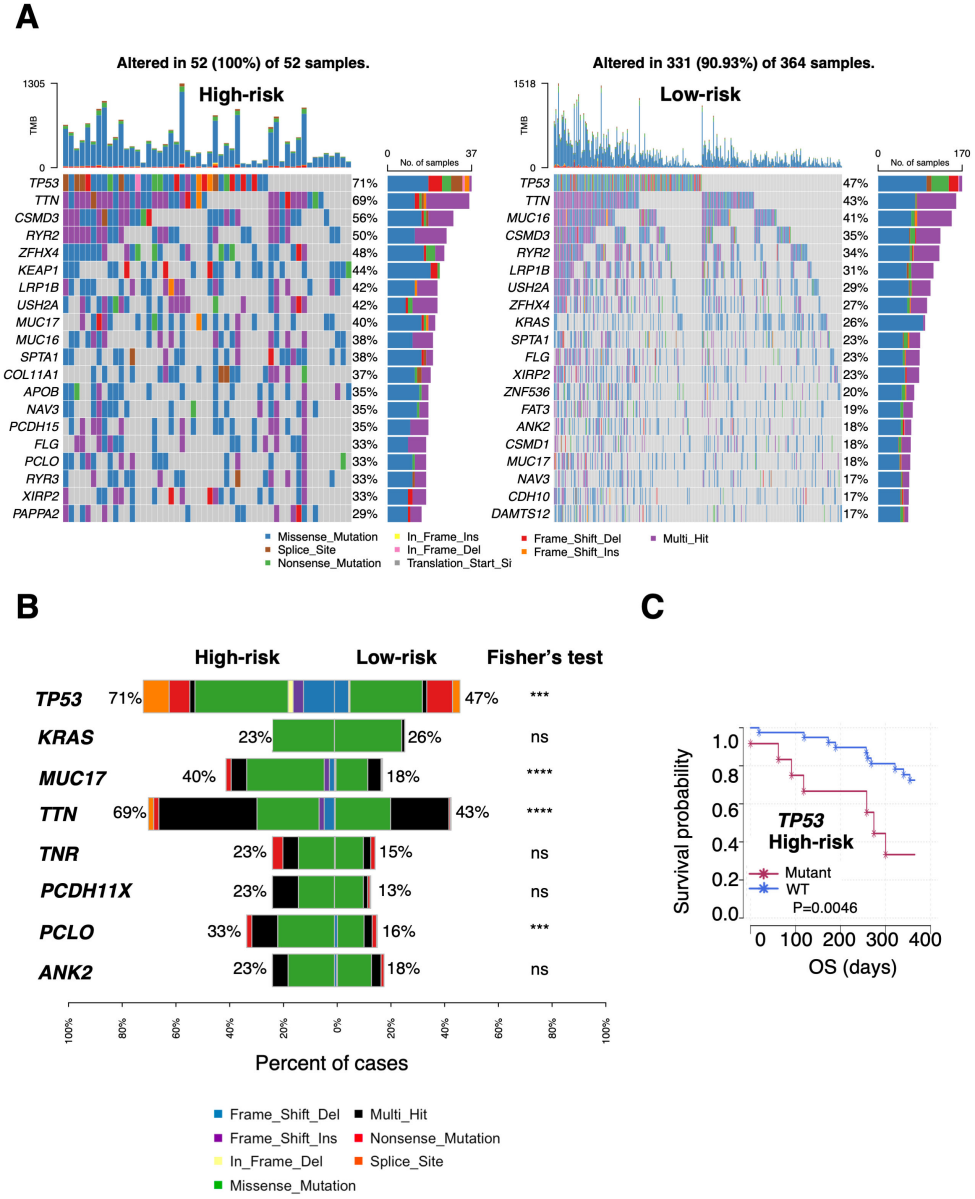
Differential landscapes of somatic mutations between the PTAAMG-Sig high- and low-risk groups in TCGA cohort. **(A)** Waterfall plot showing the somatic mutation feature of the top 20 genes in the mutation frequency in the PTAAMG-Sig high- and low-risk groups. **(B)** Frequencies of OS-associated mutations were compared between the PTAAMG-Sig high- and low-risk groups. *P*-values by Fisher’s exact test, ns, not significant; ***P < 0.001; ****P < 0.0001. **(C)** KM survival curve depicting the outcome of OS in the patients with mutant and wild-type TP53 in the PTAAMG-Sig high-risk group. *P*-values by log-rank test.

The somatic non-synonymous mutation profiles of PTAAMG-Sig high- and low-risk groups in TCGA-LUAD were analyzed. Among the top 20 genes in terms of mutation frequency in each risk group, 13 genes, including *TP53*, *TTN*, *CSMD3*, *RYR2*, *ZFHX4*, *LRP1B*, *USH2A*, *MUC17*, *MUC16*, *SPTA1*, *NAV3*, *FLG*, and *XIRP2* were shared between the two risk groups (**Figure 4A**). The rate of *TP53* (71% vs. 47%, *P* < 0.001), *MUC17* (40% vs.18%, *P* < 0.0001), *TTN* (69% vs. 43%, *P* < 0.0001), and *PCLO* (33% vs. 16%, *P* < 0.001) mutations were significantly higher in the high-risk group, whereas the difference in mutations of *KRAS* (23% vs. 26%), *TNR* (23% vs. 15%), *PCDH11X* (23% vs. 13%), and *ANK2* (23% vs. 18%) were not significant (**Figure 4B**). We further analyzed the relationship between the significantly differentially mutated genes and OS using univariate Cox regression analysis in the high- and low-risk groups. The results showed that *KRAS*, *MUC17*, *TTN*, *TNR*, *PCDH11X*, and *PCLO* mutations were positively correlated with long OS, whereas *TP53* mutation was negatively correlated with long OS in the high-risk group (HR: 3.740, Log-rank *P* = 0.0046) (**Figure 4C**). In the low-risk group, *ANK2* mutations were markedly associated with OS prognosis, but not with other factors (**Supplementary Table 4**). Notably, no significant differences occurred between the two groups in the mutation rates of the nine key PTAAMGs genes used in the signature (**Supplementary Figure 5B**).

Although the tumor mutation burden (TMB) was considered a biomarker for the prognosis of various cancers, TMB did not predict OS prognosis in the TCGA training cohort. That is, the OS of the low- and high TMB groups (divided with a cutoff value of –0.167, calculated as log2TMB/Mb, based on the optimal AUC cutoff) was not significantly different (HR = 2.503, 95% CI 0.061–10.334, log-rank *P* = 0.190) (**Supplementary Figure 7D**). However, we found that patients classified as high-risk had a greater TMB than those in the low-risk group (Wilcoxon rank sum test, *P* < 0.0001) (**Supplementary Figure 7E**). PTAAMG-Sig and TMB effectively stratified patients into high-risk with high TMB levels (N = 52), low-risk with high TMB levels (N = 323), and low-risk with low TMB levels (N = 41). Compared with low-risk with lower TMB levels, high-risk patients with higher TMB levels had poorer OS rates and showed a significant association with OS (HR = 8.302, 95% CI 1.917–35.952, log-rank *P* < 0.0001) (**Supplementary Figure 7F**). These results indicate that TMB levels were one of the factors influencing the differentiation between the high and low PTAAMG-Sig risk groups and that PTAAMA-Sig was useful for stratifying prognosis in the subgroup with higher TMB levels.

### 3.6 Association of PTAAMG-Sig with the prediction of clinical response to chemotherapy as well as ICI therapy

**Figure 5:**
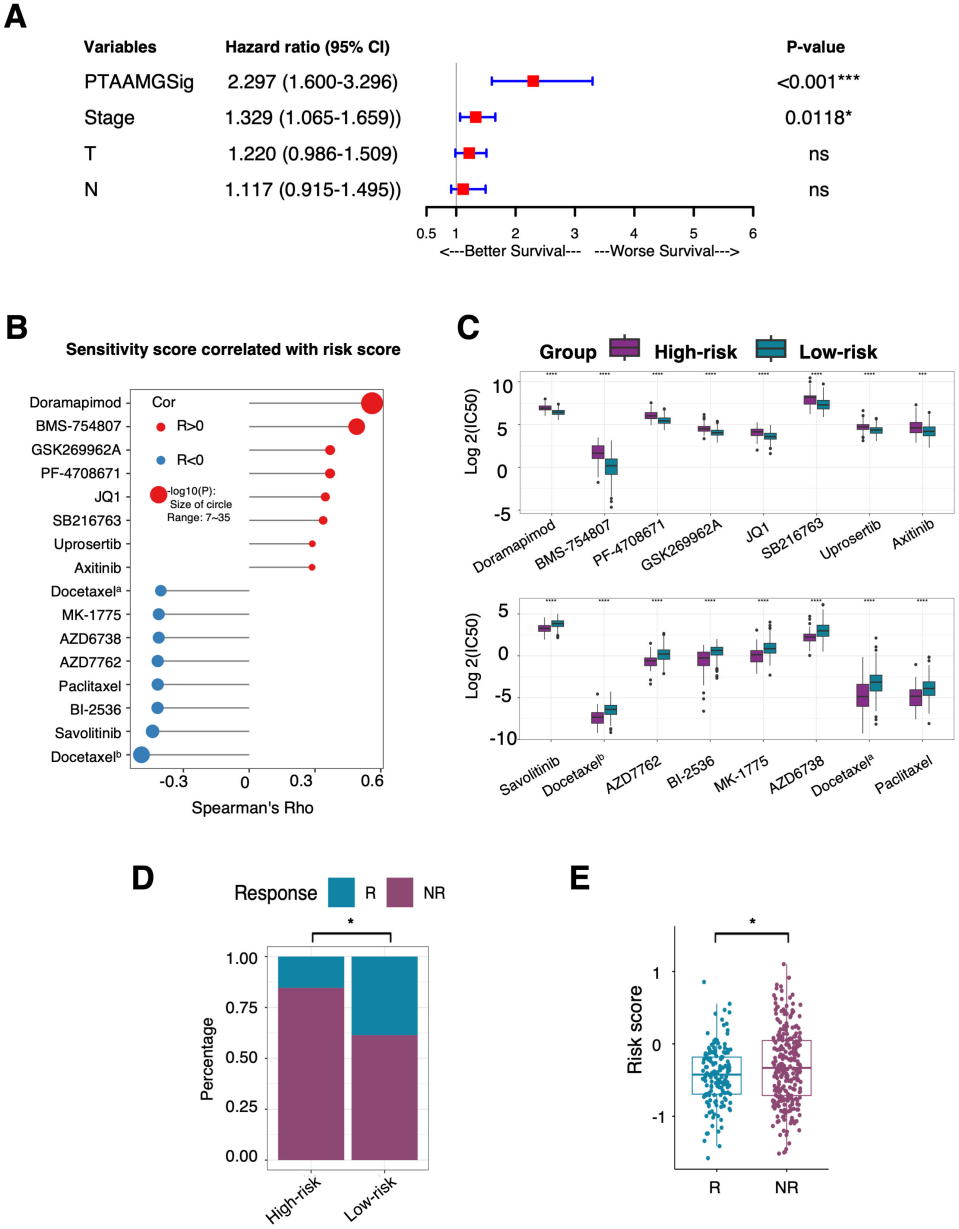
Prediction ability of the PTAAMPG-Sig on OS and response to chemotherapy and ICI therapy in TCGA-LUAD cohort. **(A)** Forest plot of the multivariate Cox regression analyses of the PTAAMPG-Sig model and important clinical factors with OS. P-values by multivariate Cox regression, ns, not significant; *P < 0.05; *** *P* < 0.001. **(B)** Spearman rank correlation analysis between the IC_50_ values and PTAAMG-Sig risk scores. X-axis: Spearman’s Rho; Y-axis: drugs; Red circle: Positive correlation; Blue circle: Negative correlation; Size of circle: −log10(P). Docetaxel^a^, docetaxel^b^ were docetaxels with different drug IDs in the GDSC2 dataset. **(C)** Relative log2(IC_50_) values predicted by OncoPredict between the PTAAMG-Sig low- and high-risk groups. Wilcoxon rank sum test, ****P < 0.0001, ***P < 0.001. Drugs were ordered based on *P*-values. **(D)** The proportion distribution of patients with TIDE predicted immunotherapeutic responses in the PTAAMG-Sig high- and low-risk groups. R, Responders; NR, Non-responders. Chi-squared test, *P < 0.05. **(E)** Boxplot showing the distribution of the PTAAMG-Sig risk scores in patients with different immunotherapeutic responses. R, Responders; NR, Non-responder. Wilcoxon rank sum test, *P < 0.05.

To explore the relationship between PTAAMG-Sig and clinical information, we combined its signature with common clinical factors from TCGA-LUAD dataset. In the univariate Cox regression analysis, sex, age, and metastasis status (M) were not significantly associated with OS. In contrast, PTAAMG-Sig, clinical stage, T, and N were significantly associated with OS (*P* < 0.05) (**Table 2**). Multivariate Cox regression analysis revealed that PTAAMG-Sig (HR: 2.297, 95% CI 1.600–3.296, *P* < 0.001) and clinical stage (HR: 1.329, 95% CI 1.065–1.659, *P* = 0.0118) were independent predictors of OS (**Figure 5A**). Smoking is an important risk factor for lung cancer. We divided the patients into non-smokers (less than 100 cigarettes smoked in their lifetime) and smokers (including current smokers and current reformed smokers). Tobacco smoking history did not predict the OS prognosis. OS was not significantly different between smokers and non-smokers (HR = 0.832, 95% CI: 0.544–1.174, log-rank *P* = 0.400) (**Supplementary Figure 8A**). Smokers and non-smokers showed no significant difference in the PTAAMG-Sig risk distribution (chi-square test, NS: *P* > 0.05) (**Supplementary Figure 8B**). In combination with PTAAMA-Sig, we found that in smokers, high-risk patients showed a significantly worse OS outcome than low-risk patients (HR = 3.399, 95% CI: 2.278–5.073, log-rank *P* < 0.0001) but not in non-smokers (HR = 2.893, 95% CI: 0.844–9.920, log-rank *P* = 0.0771) (**Supplementary Figures 8C, D**).

**Table 2:** Cox regression analysis of PTAAMG-Sig and clinical factors in TCGA cohort.

Variables	Univariate Cox regression	
	P-value	HR (95% CI)
Sex*	0.995	1.001 (0.729−1.375)
**Age**	0.477	1.006 (0.989−1.023)
**M**	0.056	1.625 (1.334−1.980)
**T**	1.38E−06	1.677 (1.396−2.015)
**N**	3.25E−08	1.792 (0.985−3.260)
**Stage**	1.99E−10	1.625 (1.399−1.887)
**PTAAMG-Sig**	7.42E−09	2.718 (1.937−3.815)

Stage All stage data were scored using the American Joint Commission on Cancer staging system. *Sex: univariate Cox regression analysis was used as a categorical variable. Ref. group: female.

As PTAAMG-Sig could predict the prognosis of the LUAD patients in three independent cohorts with different treatment modalities, we further investigated the relationship between PTAAMG-Sig and the prediction of response to chemotherapy and ICI therapy. The drug sensitivity prediction of TCGA-LUAD patients was performed on the “oncoPredict” algorithm utilizing the data from the GDSC v2 database. Spearman rank correlation analysis demonstrated that the predicted IC_50_ value of doramapimod (p38 MAPK inhibitor), BMS-754807 (IGF-1R/InsR inhibitor), GSK269962A (ROCK inhibitor), PF-4708671 (cell-permeable S6K1 inhibitor), JQ1 (BET bromodomain inhibitor), SB216763 (ATP-competitive GSK-3 inhibitor), uprosertib (AKT inhibitor), and axitinib (multi-target inhibitor for VEGFR1, VEGFR2, VEGFR3, and PDGFRβ) were positively correlated with the PTAAMG-Sig risk score, indicating that the PTAAMG-Sig high-risk group had a relatively higher resistance to these therapeutics than the low-risk group. Conversely, the PTAAMG-Sig high-risk group showed higher sensitivity to docetaxel (microtubule depolymerization inhibitor, docetaxel^a^ and docetaxel^b^), savolitinib (c-MET inhibitor), BI-2536 (PLK1 and BPD4 inhibitor), paclitaxel (microtubule stabilizer), AZD7762 (ATP-competitive Chk inhibitor), AZD6738 (ATR kinase inhibitor), and MK-1775 (Wee1 inhibitor) than the low-risk group (**Figures 5B, C**).

Because genomic mutations can alter tumor immune profiles and response to immunotherapy, we further evaluated the predictive capability of PTAAMG-Sig for response to ICI therapy in TCGA-LUAD patients using the TIDE algorithm. TIDE predicted that 84.6% of TCGA-LUAD patients with PTAAMG-Sig high-risk scores were non-responders to ICI treatment, whereas 61.4% of LUAD patients with low-risk scores were non-responders, and the difference was significant (chi-square test, *P* < 0.01) (**Figure 5D**). This indicates that immunotherapy was more likely to be successful in patients in the PTAAMG-Sig low-risk group. In fact, TIDE-predicted non-responders showed significantly higher PTAAMG-Sig risk scores than responders (Wilcoxon rank sum test, *P* < 0.05) (**Figure 5E**).

### 3.7 Differences in the gene expression profile between the PTAAMG-Sig high- and low-risk groups

To understand the functional significance of the PTAAMG-Sig signature, stromal and immune scores were analyzed using the ESTIMATE algorithm. The PTAAMG-Sig low-risk group showed significantly higher stromal and immune scores than the high-risk group, indicating that the tumors in the low-risk group had a TME with high stromal and immune activity (**Supplementary Figure 9A**). The DEGs between the high- and low-risk groups identified using PTAAMG-Sig were subjected to GSEA for the GO dataset. We identified that the “Metabolism of amino acids and derivatives” pathway was positively enriched in the PTAAMG-Sig high-risk group (NES = −1.691, FDR < 0.001) (**Supplementary Figure 9B**). Furthermore, in the high-risk group, pathways related to “DNA replication”, “cell cycle checkpoints”, and “apoptosis” were significantly positively enriched. In the low-risk group, the “SLC-mediated transmembrane transport” pathway was significantly enriched, which is possibly related to altered amino acid transmembrane transport and metabolism. “PD-1 signaling”, “Phosphorylation of CD3 and TCR zeta chains”, and “Cytokine-cytokine receptor interaction” pathways correlated with immune activity and ICIs treatment process were also significantly highly enriched in the low-risk group (**Supplementary Figure 9C**, **Supplementary Table 5**). These results demonstrate the predictive capability of this signature for ICIs therapy.

### 3.8 The possibility of PTAAMG-Sig to predict clinical response to ICIs therapy

**Figure 6:**
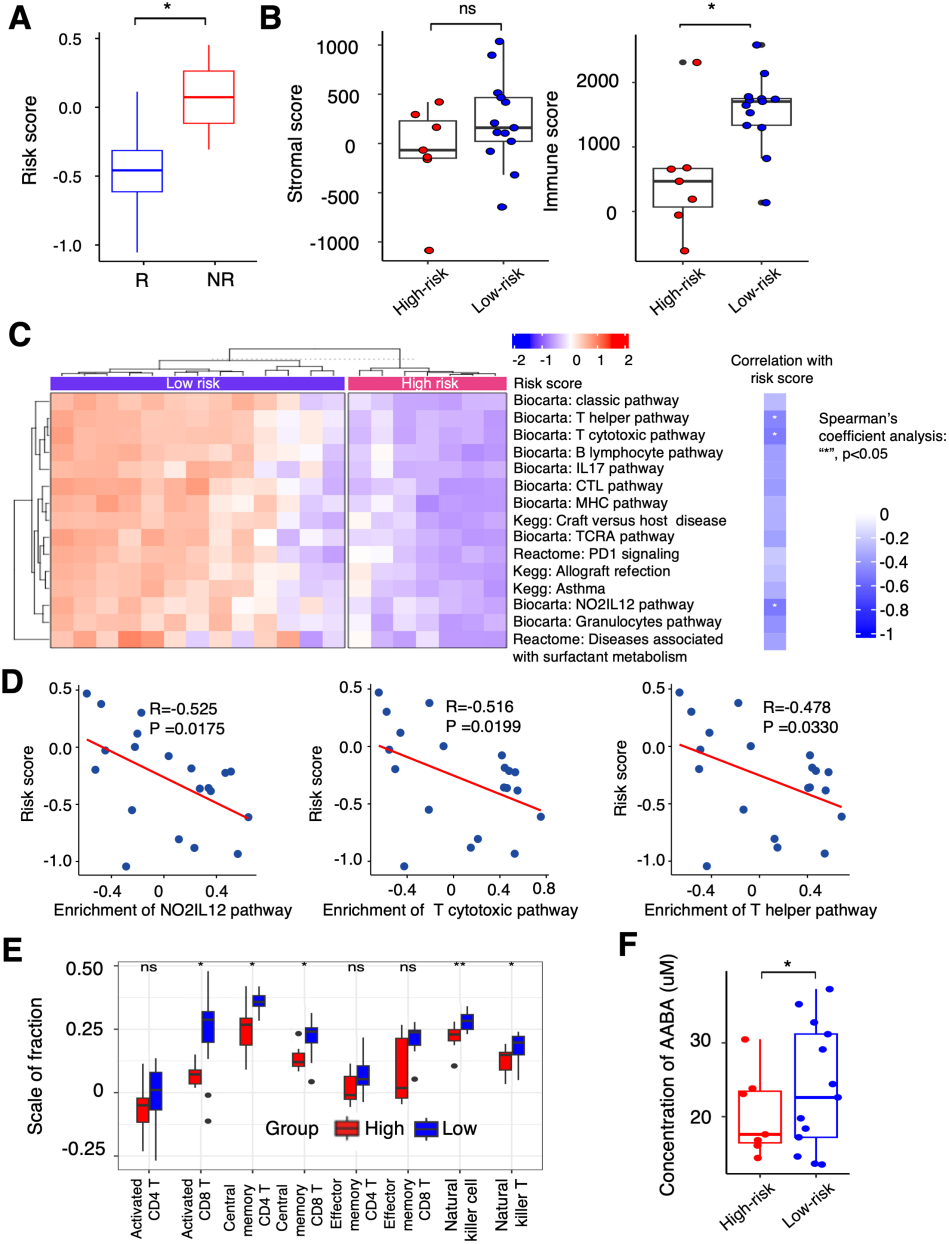
Differential landscapes of immune infiltrations between the PTAAMG-Sig high- and low-risk groups and the correlation with the concentration of PFAAs in blood in the KCC-ICI cohort. **(A)** A boxplot showing the distribution of the PTAAMG-Sig risk scores in patients with different immunotherapeutic responses. R: Responders, including Stable Disease (SD) or Partial Response (PR); NR: Non-responders, Progressive Disease (PD). Wilcoxon rank sum test, *P< 0.05. **(B)** Boxplots depicting stromal and immune scores using the ESTIMATE algorithm in the PTAAMG-Sig high- and low-risk groups. Wilcoxon rank sum test, ns: not significant; *P< 0.05. **(C)** Left figure: Heatmap of significantly differentially enriched pathways for signature genes from Biocarta, Kegg, and Reactome databases based on GSVA between the PTAAMG-Sig high- and low-risk groups. Blue: low enriched; Red: high enriched. Right figure: Heatmap of the correlations between significantly enriched pathways and the PTAAMG-Sig risk scores. Spearman’s coefficient analysis, *P < 0.05. Blue: negative correlation. **(D)** Scatter plots presenting the correlations between the PTAAMG-Sig risk scores with the enrichment scores of the “NO2IL12 pathway,” “T cytotoxic pathway,” and “T helper pathway” from the Biocarta database. Spearman’s correlation analysis. *P < 0.05. **(E)** Relative abundance of activated CD4 T cells, activated CD8 T cells, central memory CD4 T cells, central memory CD8 T cells, effect memory CD4 T cells, effect memory CD8 T cells, natural killer cells, and natural killer T cells calculated using ESTIMATE in the PTAAMG-Sig high- and low-risk groups. Wilcoxon rank sum test, **P < 0.01, *P < 0.05. **(F)** Boxplot presenting concentrations of α-ABA in the PTAAMG-Sig high- and low-risk groups. Wilcoxon rank sum test, *P< 0.05.

The relationship between the PTAAMG-Sig risk scores and OS in the KCC-ICI cohort treated with combined chemotherapy and ICIs therapy is shown in **Figure 3**. We further estimated the ability of this signature to predict patient responses to this therapy. The responders in the treatment group had significantly lower PTAAMG-Sig risk scores than non-responders (Wilcoxon rank sum test, *P* < 0.05) (**Figure 6A**).

Transcriptome analysis of the tumor FFPE specimens in this cohort demonstrated that the PTAAMG-Sig low-risk group exhibited a significantly higher immune score than the high-risk group. However, the stromal scores did not differ, indicating elevated immune activity in the low-risk group (Wilcoxon rank sum test, *P* < 0.05) (**Figure 6B**). To address the apparent differences in immune function between them, we performed GSVA using the Biocarta, KEGG, and Reactome databases. The PTAAMG-Sig low-risk group showed several significantly enriched pathways related to immune cell regulation, including T cell activation, inflamed status, and some other immune-related pathways, such as “Classic pathway”, “Graft versus host disease”, “Allograft rejection”, and “Diseases associated with surfactant metabolism” (**Figure 6C**, left). The heatmap exhibits Spearman’s rank correlations between the PTAAMG-Sig risk scores and the enrichment scores of the significantly altered pathways (**Figure 6C**, right). Among the pathways, the scatter plot analysis identified the “NO2-IL12 pathway” (R = −0.525, *P* = 0.0175), “T cytotoxic pathway” (R = −0.516, *P* = 0.0199), and “T helper pathway” (R = −0.478, *P* = 0.0330) as significantly negatively correlated pathways with the PTAAMG-Sig risk scores (**Figures 6C, D**). Subsequently, we investigated the differences in T, NK, and NK-T cells between the PTAAMG-Sig low- and high-risk groups by determining the infiltrating immune cell population in the TME, deconvoluted from the results of RNA sequencing. Activated CD8 T, central memory CD4 T, central memory CD8 T, NK, and NK-T cells were significantly increased in the low-risk group (Wilcoxon rank sum test, *P* < 0.05) (**Figure 6E**).

### 3.9 Relationship between concentrations of PFAAs and PTAAMG-Sig

The PTAAMG-Sig model was developed from the expression profiles of genes related to amino acid metabolism pathways, based on their significance in tumor biology and immunity. We examined the relationship between PFAA concentration and PTAAMG-Sig risk scores in a KCC-ICI cohort treated with combined chemotherapy and ICI therapy. AABA was the only amino acid whose concentration differed significantly between the high- and low-risk PTAAG-Sig groups, with a lower concentration in the high-risk group (Wilcoxon rank sum test, *P* < 0.05) (**Figure 6F**, **Supplementary Figure 10A**). To address the target factors, we performed Spearman rank correlation analysis, and the results showed that the concentration of AABA was significantly negatively correlated with *MIF* gene expression levels in TME (R=-0.459, *P*=0.0419) (**Supplementary Figures 10B, C**).

## 4 Discussion

Prognostic gene sets obtained by screening large datasets of tumors can provide direct and accurate information. Although amino acid metabolism can be estimated directly by mass spectrometry, the expression profiles of genes involved in this metabolic process may provide a comprehensive view of the regulatory mechanisms and pathways involved in amino acid metabolism. Recently, researchers have focused on genes involved in amino acid metabolism pathways and their impact on patient prognosis by using public datasets (23, 24). In LUAD, one study provided a signature derived from RNA sequencing data of amino acid metabolism-related genes to predict prognosis. This signature consisted of *CPS1*, *AZIN2*, *GNMT*, *PSPH*, *RIMKLA*, and *S*MOX (25). Although the signature successfully predicted prognosis in TCGA_LUAD and two other GEO datasets, a comprehensive multi-omics understanding was lacking. In this study, based on transcriptional profiling data from several cohorts, including our original KCC-ICI cohort, we developed and validated a novel signature related to amino acid metabolism to predict cancer prognosis. Furthermore, multi-omics analyses and bioinformatics approaches were applied to explore how somatic mutations, immunological landscapes, and PFAA profiles differed between high- and low-risk groups.

Notably, our study of the KCC-ICI cohort suggested that PTAAMG-Sig has the potential to predict the clinical response of patients to chemotherapy combined with immune checkpoint blockade therapy and also OS with a relatively high AUCmax at 0.842 value. To understand its predictive mechanism, we performed transcriptome analysis of tumor tissues and investigated the correlation between PTAAMG-Sig and deduced immune cell infiltration or gene expression profiles. The PTAAMG-Sig low-risk group had higher infiltration of immune cells, as estimated by the immune score, which included activated and central memory CD8 T cells, central memory CD4 T cells, NK cells, and NK-T cells. Gene expression profiles were significantly enriched in immune regulatory pathways mediated by relevant T cell and NK cell activation. Additionally, we found that the gene expression profile in the low-risk group showed higher enrichment in the PD1 signaling pathway in the reactome, which provided a direct basis for a better response to ICI therapy in this group. These findings provide evidence for the effectiveness of immunotherapy in the low-risk PTAAMG-Sig group.

Recently, we reported the clinical significance of PFAAs profile and their metabolites in NSCLC patients treated with PD-1 inhibitors (20). To know the PFAAs’ alteration associated with PTAAMG-Sig, we screened PFAAs profile in the KCC-ICI cohort to determine PFAAs’ alterations associated with PTAAMG-Sig. The results showed that the pretreatment plasma-free AABA concentration was significantly higher in the PTAAMG-Sig low-risk group than in the high-risk group. AABA is produced through cysteine biosynthesis or metabolism of methionine, threonine, serine, and glycine, as a byproduct (26, 27). AABA is a non-proteinogenic amino acid, and plasma AABA levels are reported to be associated with the progression of sepsis (26, 28). Furthermore, AABA improves the survival of septic mice and reduces disease severity in experimental colitis by inhibiting the polarization and function of M1 pro-inflammatory macrophages (29). Our study found a significant negative correlation between the expression level of *MIF* and AABA concentration. Previous research has reported that MIF deficiency and treatment with the small-molecule MIF inhibitor 4-IPP contribute to the restoration of immunosuppressive tumor progression of tumor-associated macrophages to M1-like polarization characteristics (30, 31). MIF was one of the key PTAAMGs identified in our study. The present study’s findings suggest that the elevated plasma AABA in the PTAAMG-Sig low-risk group was associated with the M1 polarization-mediated inflammatory TME and prognostic outcome of ICI treatment in LUAD patients.

*TP53* mutations are prevalent in tumor development, not only diminishing the tumor suppressive function of the wild-type protein but also conferring pro-tumor activity (32). p53 plays a vital role in regulating metabolic processes both in tumor and non-tumor cells (33). However, its specific regulatory mechanism in amino acid metabolism has not been fully addressed. We observed that the rate of *TP53* mutations was significantly higher in the PTAAMG-Sig high-risk group than in low-risk group and that the high-risk group with *TP53* mutations had the worst OS prognosis. Therefore, the combined effects of PTAAMG-Sig and *TP53* mutation status in predicting the prognosis of LUAD patients should be considered. When we used the GDSC v2 database to predict the efficacy of chemotherapeutic drugs, the high- and low-risk groups showed a robust diversity of sensitive drug groups. For example, high-risk groups associated with a higher frequency of *TP53* mutations were predicted to have significant sensitivity to the c-Met inhibitor, savolitinib. Several studies have indicated that specific *TP53* mutations can impact downstream signaling pathways, including c-Met signaling (34). These mutations induced c-Met expression or elevated its activity, making cancer cells more dependent on the c-Met pathway for survival and growth (35). Consequently, the possibility occurs that the PTAAMG-Sig high-risk group patients with *TP53* mutation, showing the worst prognosis, benefit from c-Met inhibitors. This provides crucial insights into the relationship between PTAAMG-Sig and perturbed p53 function.

The established novel signature, PTAAMG-Sig, comprises nine genes related to amino acid metabolism. Among these nine genes, *ALDH2*, *ACAD8*, *HDC*, and *TYRP1* were upregulated in the low-risk group. *ALDH2* encodes aldehyde dehydrogenase 2 protein found in mitochondria that is involved in ethanol metabolism (36). In our enrichment analysis, ALDH2 is enriched in three of the four specific pathways related to amino acid metabolism: the histidine metabolic pathway; tryptophan metabolic pathway; and valine, leucine, and isoleucine metabolic pathway. ALDH2 deficiency activates oncogenic pathways via extracellular vesicles enriched in oxidized DNA, promoting alcohol-associated hepatocellular carcinoma (37). ALDH2 also influences the clearance of endogenous aldehyde 4-HNE produced by ROS-mediated peroxidation reactions. 4-HNE frequently causes a hotspot mutation of *TP53* at codon 249 in the DNA-binding domain in hepatocellular cancer (38). In LUAD cells, elevated ALDH2 activity with its chemical agonist Alda-1 inhibited the stemness, proliferation, and migration and reduced DNA damage (39). Furthermore, ALDH2 expression in tumor cells is significantly and positively correlated with the infiltration of immune cells, including CD4+ T cells, CD8+ T cells, neutrophils, B cells, and macrophages in various tumor types (40). These tumor suppressive functions of ALDH2 are consistent with the established PTAAMG-Sig, in which *ALDH2* is associated with a good patient prognosis. Acyl-CoA dehydrogenase family member 8 (ACAD8) is an isobutyryl-CoA dehydrogenase that plays a role in the catabolism of branched-chain amino acids, including valine. Limited information is available on the role of ACAD8 in the TME, which is regarded as a tumor-associated fibroblast-related gene associated with good survival outcomes (41). Similar to ALDH2, ACAD8 has been reported to be associated with a favorable prognosis in LUAD, which is consistent with the signature that we developed. However, TGF-β1 derived from histidine carboxylase (HDC)-expressing myeloid-derived suppressor cells (MDSC) promotes epithelial-mesenchymal transition in metastatic LUAD (42). Cytokines/chemokines secreted by tumor tissues are responsible for the expansion of HDC^+^ MDSC and their transport to breast tumors (43), in contrast to our signature.

Conversely, expression of *PPAT*, *MIF*, *GCLC*, *PSPH*, and *KYNU* among the nine genes was elevated in the high-risk group of the signature associated with the poor prognosis of the patients. Because *KYNU* was the only gene whose expression was significantly and independently correlated with prognosis in multiplex Cox regression analysis among the nine genes of the signature, it piqued our interest as a risk factor. *KYNU* encodes kynureninase, which catalyzes kynurenine (Kyn), a tryptophan (Trp) metabolite. *KYNU* overexpression has been linked to the development and prognosis of several cancers (44–47). The Kyn-mediated Trp-Kyn-arylhydrocarbon receptor AhR pathway, which promotes immune cell differentiation and apoptosis, is also one of the immune escape mechanisms of cancer cells in inflammatory tumors or so-called hot tumors with lymphocyte infiltration (48). Recent research further linked the mutation-activated NRF2 pathway in LUAD to the upregulation of *KYNU* in TME, resulting in immunosuppression and poor prognosis of the patients (49). These findings may support that the *KYNU* was involved in the established PTAAMG-Sig as a poor prognostic parameter. Phosphoribosyl pyrophosphate aminotransferase (PPAT), a *de novo* purine biosynthetic enzyme, regulates lung cancer cell proliferation and invasion by upregulating pyruvate kinase activity (50). Activated T cells release macrophage migration inhibitory factor to suppress glucocorticoid-mediated production of IL-2 and IFN-γ, which promotes lung cancer cell proliferation and the Warburg effect (51). Enforced expression of the glutamate-cysteine ligase catalytic subunit is an effective way to promote glutathione synthesis. GCLC expression is also related to increased cisplatin resistance in human NSCLC xenografts *in vivo* (52). Phosphoserine phosphatase (PSPH) is a key factor in the malignant progression of lung cancer cells and cancer drug resistance (53). In addition to *KYNU*, genes encoding these proteins may play positive roles in cancer development and are associated with poor patient prognosis.

The variation in the purity of TCGA samples was reported to affect the prediction of prognosis signature; therefore, we further calculated the tumor purity score in the TCGA cohort using mathematical algorithms (54). After adjusting for purity, we investigated the association between the PTAAMG-Sig risk scores and OS outcomes. PTAAMG-Sig also had a powerful effect on OS prediction (C-index = 0.673, log-rank *P* = 1e–08). Given that this tumor purity score has not yet been experimentally validated, this concept requires further investigation.

In conclusion, based on the genes involved in amino acid metabolism, we developed PTAAMG-Sig, which showed promise for the prediction of OS as well as chemotherapy and immunotherapy responses in LUAD patients. Our original cohort and a GEO cohort demonstrated the potential for the signature to be applied in patients treated with ICIs. Multi-omics characterization showed that PTAAMG-Sig was associated with *TP53* mutation, immune cell alteration in the TME, and AABA concentration in the blood. Notably, the signature was a significant independent factor for OS prediction compared to existing ones. In addition, PTAAMG-Sig was useful for prognostic stratification in the subgroup with higher TMB or PD-L1 expression and smoking history. Our study provides a strong basis for the development of new therapeutic strategies and personalized treatment options for patients with LUAD. However, this study has some limitations; in particular, our cohort of patients with ICI therapy was small, and we must further validate or refine the established signature with a larger cohort in the future. The data from GEO (GSE31210, GSE50081, GSE68465) used for our validation cohorts were generated using different platforms of comprehensive transcriptome analysis than those used for the TCGA cohort; thus, the GEO cohorts may not be comparable to the TCGA cohort. Transcriptomic profile-based bioinformatic analysis was carried out for estimation of immune cell distribution and activity; hence, the relationship between our signature and immune cell alteration still warrants further experimental validation. The specific causality relationship of our PTAAMG-Sig and other integrated multi-omics factors needs further investigation.

## Data Availability

The dataset presented in this study can be found in the online repository DDBJ (DNA Data Bank of Japan) under the GEAAccession number E-GEAD-883: https://ddbj.nig.ac.jp/public/ddbj_database/gea/experiment/E-GEAD-000/E-GEAD-883/.
