# Supplementary material for: Multi-omics analysis-based clinical and functional significance of a novel prognostic and immunotherapeutic gene signature derived from amino acid metabolism pathways in lung adenocarcinoma
Source: Front Immunol. 2024 Dec 13;15:1361992. doi: 10.3389/fimmu.2024.1361992 (PMC11671776; doi:10.3389/fimmu.2024.1361992)
Supplement: Supplementary file 1 [file Presentation1.pdf]

Supplementary Material

1 Supplementary Tables

Supplementary Table 1. A list of Amino acid metabolism-related genes (AAMGs).

Gene symbol										
GOT1	*PRDM7*	*AGXT*	*ACADM*	*SETD1B*	*LAP3*	*PLOD3*	*PNMT*	*AGMAT*	*PHYKPL*	*LRTOMT*
ABHD14AACY1	*PRODH*	*ALDH2*	*ACADS*	*SETD2*	*LDHAL6A*	*PRDM9*	*SHMT2*	*ALDH8A1*	*PRDM2*	*NAT8L*
AGXT2	*SRR*	*ALDH3A2*	*ACADSB*	*SETD7*	*LDHC*	*PRODH2*	*ALDH7A1*	*ASL*	*SMS*	*PYCR1*
AHCY	*ALDH18A1*	*ALDH3B1*	*ACAT1*	*SETDB1*	*MAOA*	*ALAS1*	*AOC1*	*BBOX1*	*TDO2*	*RIMKLA*
AHCYL1	*ALDH1B1*	*ALDH3B2*	*ACAT2*	*SETDB2*	*MAOB*	*ALDH6A1*	*ARG1*	*BCKDHA*	*AANAT*	*SAT1*
ALAS2	*ALDH4A1*	*CKMT2*	*ACMSD*	*SETMAR*	*MAT2A*	*AMT*	*ASH1L*	*CTH*	*AASS*	*SMYD3*
ALDH1A3	*ALDH5A1*	*DDC*	*ADH1A*	*SMYD1*	*MAT2B*	*APIP*	*ASS1*	*CYP1B1*	*ACY1*	*SRM*
BHMT	*CAMKMT*	*FAHD1*	*ADH5*	*SUV39H2*	*NAGS*	*ASRGL1*	*AZIN2*	*DBT*	*ADH1C*	*TMLHE*
BPGM	*CNDP2*	*GAD1*	*ALDH3A1*	*TPH2*	*PYCR3*	*BHMT2*	*BCAT2*	*HIBCH*	*ADH4*	*TPH1*
DNMT1	*CPS1*	*GCLC*	*AMD1*	*FTCD*	*SARDH*	*CAT*	*CBS*	*IDO1*	*AHCYL2*	*TPO*
DOT1L	*CYP1A2*	*GCLM*	*AOC3*	*GAMT*	*SMYD2*	*CKM*	*CKMT1A*	*IL4I1*	*AOC2*	*PIPOX*
ECHS1	*GCDH*	*GLUL*	*ASNS*	*AADAT*	*TST*	*CLYAT*	*CNDP1*	*IL4T1*	*ARG2*	*PRDM6*
EHMT1	*GCSH*	*GNMT*	*CAD*	*ABAT*	*TYR*	*COLGALT1*	*COLGALT2*	*MCCC1*	*AUH*	*TH*
EHMT2	*GLUD2*	*GOT2*	*CARNMT1*	*ACSF3*	*TYRP1*	*DHTKD1*	*EHHADH*	*MCCC2*	*CBSL*	
GFPT1	*GLYCTK*	*HMGCS2*	*CHDH*	*ADH1B*	*UROC1*	*DLST*	*GLDC*	*PHGDH*	*CDO1*	
HMGCL	*GPT*	*KYAT3*	*DBH*	*AMDHD1*	*ACAA2*	*EZH2*	*GLS2*	*PPAT*	*CKMT1B*	
KMO	*HMGCS1*	*KYNU*	*EZH1*	*AOX1*	*ADH6*	*GLS*	*HADHB*	*PSPH*	*COMT*	
MCEE	*HSD17B10*	*L3HYPDH*	*FOLH1*	*ASPA*	*DDO*	*GPT2*	*HDC*	*SETD1A*	*DCT*	
MDH2	*KMT2C*	*MAT1A*	*GAD2*	*CARNS1*	*DLD*	*GRHPR*	*MDH1*	*SMOX*	*DNMT3B*	
MIF	*KYAT1*	*NIT2*	*GATM*	*CKB*	*DNMT3A*	*GSS*	*SDS*	*TAT*	*GLUD1*	
NOS3	*MMUT*	*NOS1*	*GCAT*	*DMGDH*	*ENOPH1*	*GSTZ1*	*SDSL*	*ALDH9A1*	*HNMT*	
NSD1	*MRI1*	*PGAM1*	*IDO2*	*FAH*	*HMGCLL1*	*HAAO*	*SHMT11*	*ASMT*	*HOGA1*	
NSD3	*MTR*	*PLOD2*	*KMT2D*	*GFPT2*	*OAT*	*HADHA*	*ADH7*	*BCAT1*	*HPD*	
ODC1	*NOS2*	*PSAT2*	*LDHA*	*HGD*	*OTC*	*HAL*	*ADI1*	*BCKDHB*	*IVD*	
OXCT1	*NSD2*	*SUV39H1*	*LDHB*	*HIBADH*	*OXCT2*	*HYKK*	*ADSL*	*CYP1A1*	*KMT2B*	
P4HA1	*PCCA*	*AACS*	*PYCR2*	*KMT2A*	*P4HA2*	*MPST*	*ADSS1*	*DAO*	*KMTSA*	
P4HA3	*PCCB*	*ACAA1*	*RIMKLB*	*KMT2E*	*PAH*	*MTAP*	*ADSS2*	*HADH*	*KMTSC*	
PLOD1	*PSAT1*	*ACAD8*	*SAT2*	*KMTSB*	*PGAM2*	*PGMA4*	*AFMID*	*INMT*	*LDHAL6B*	

Supplementary Table 2. A list of AAMGs associated with OS.

Gene	Hazard Ratio	P-value
PPAT	1.421	0.002
PSPH	1.287	0.004
AGMAT	1.271	0.016
MIF	1.226	0.015
GCLM	1.170	0.035
KYNU	1.160	0.002
GCLC	1.126	0.011
ADH1B	0.892	0.003
AOC3	0.878	0.042
MAOB	0.840	0.012
INMT	0.828	0.001
ALDH2	0.806	0.005
TYRP1	0.793	0.008
ACAD8	0.775	0.009
HDC	0.749	0.003
ASPA	0.690	0.049

Sixteen genes significantly associated with the OS of TCGA-LUAD patients were analyzed using univariate Cox regression analysis.

Supplementary Table 3. Cox regression analysis of clinical factors in KCC-ICI cohort.

Variables	Univariate Cox regression	
	P-value	HR (95% CI)
***Sex**	0.569	0.498 (0.0451–5.49)
**Age**	0.165	0.917 (0.81–1.04)
**Stage**	0.245	3.74 (0.405–34.6)

All stage data were scored using the American Joint Commission on Cancer (AJCC) Staging System.
*Sex: The univariate COX regression analysis was used as a categorical variable. Ref. group: female.

Supplementary Table 4. Univariate Cox regression of somatic mutations in the PTAAMGSig high- and low-risk groups.

High-risk group					Low-risk group				
Gene	P-value	HR	N (WT)	N (Mutant)	Gene	P-value	HR	N (WT)	N (Mutant)
TP53	0.00463	3.74	15	37	***ANK2***	0.0391	0.16	297	67
KRAS	0.0135	0.319	40	12	***CDH10***	0.0541	0.176	302	62
MUC17	0.0214	0.256	31	21	***COL11A1***	0.0603	2.15	304	60
TTN	0.0325	0.368	16	36	***MUC16***	0.0711	1.97	215	149
TNR	0.035	0.152	40	12	***ZNF804A***	0.0799	0.201	310	54
PCDH11X	0.0432	0.161	40	12	***NAV3***	0.131	0.347	301	63
PCLO	0.0482	0.304	35	17	***ERICH3***	0.14	1.95	315	49
USH2A	0.0567	0.352	30	22	***TNR***	0.144	1.87	309	55
COL5A2	0.079	0.287	39	13	***TP53***	0.219	1.59	194	170
ZNF831	0.0858	0.203	40	12	***ZFHX4***	0.237	0.562	265	99
FLG	0.0868	0.352	35	17	***PAPPA2***	0.249	1.69	309	55
FAT3	0.100	0.31	40	12	***PTPRD***	0.251	0.44	313	51
ZNF804A	0.148	0.352	40	12	***RYR1***	0.264	1.66	311	53
DNAH8	0.181	0.38	40	12	***KEAP1***	0.287	0.466	313	51
ANK2	0.202	0.396	40	12	***FAT4***	0.307	0.481	312	52
LRRC7	0.221	0.41	41	11	***MUC17***	0.31	0.543	299	65
CSMD2	0.231	0.417	40	12	***DMD***	0.31	0.483	315	49
XIRP2	0.234	0.511	35	17	***RP1L1***	0.323	1.57	309	55
ERICH3	0.237	0.479	38	14	***APOB***	0.329	1.56	308	56
ADGRG4	0.259	0.495	37	15	***NPAP1***	0.366	1.51	307	57

Supplementary Table 5. Reactome or KEGG GSEA pathway enrichment of differentially expressed genes between PTAAMG-Sig low- and high-risk groups.

ID	Description	NES	P-value	Q-value
**R-HSA-69306**	DNA Replication	−2.657	<0.001	<0.001
**R-HSA-69620**	Cell Cycle Checkpoints	−2.798	<0.001	<0.001
**R-HSA-109581**	Apoptosis	−1.727	<0.001	<0.001
**R-HSA-389948**	PD-1 signaling	1.710	0.006	0.018
**R-HSA-202427**	Phosphorylation of CD3 and TCR zeta chains	1.890	0.000	0.001
**R-HSA-425407**	SLC-mediated transmembrane transport	1.347	0.008	0.023
**Hsa04060**	Cytokine-cytokine receptor interaction	1.468	0.001	0.008

2 Supplementary Figures

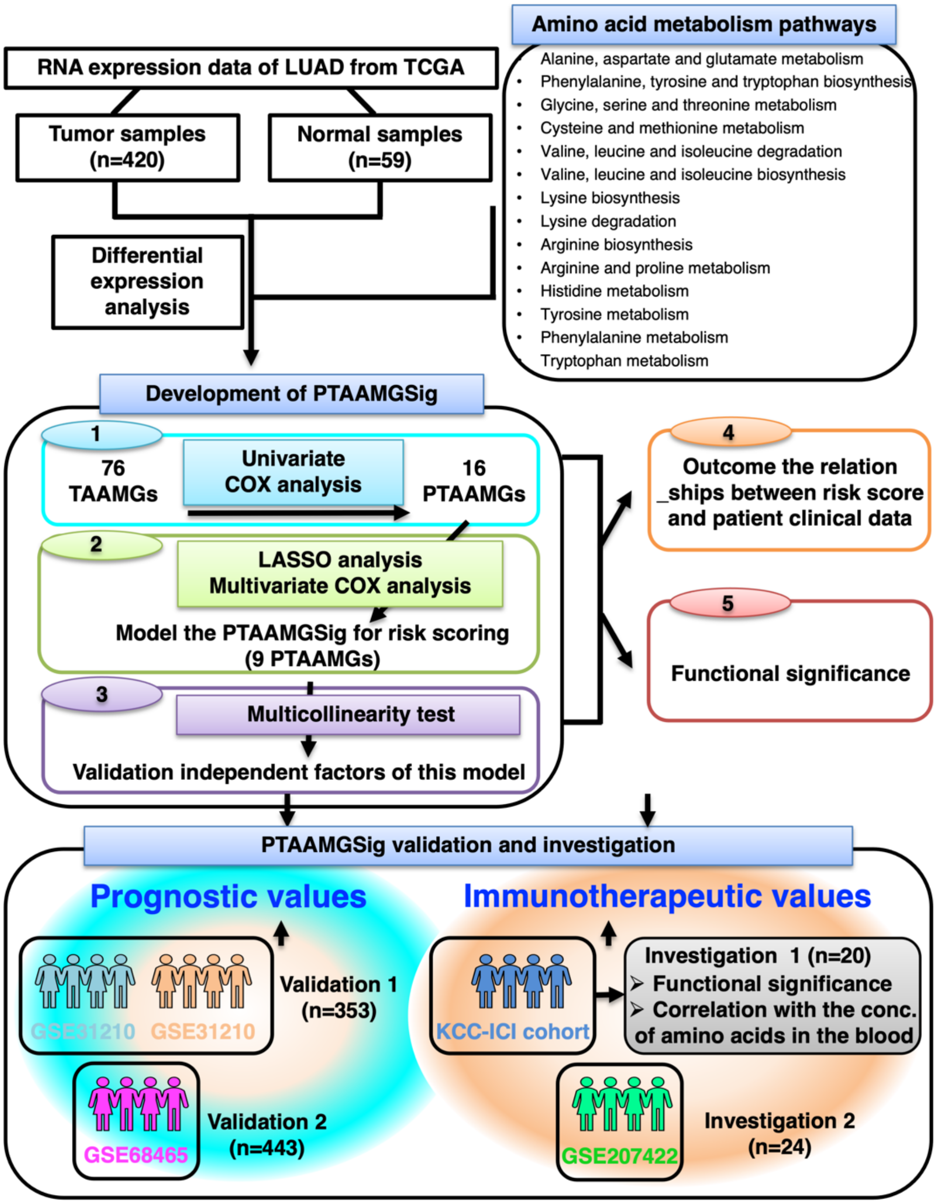

Supplementary Figure 1. Schematic overview of study design.

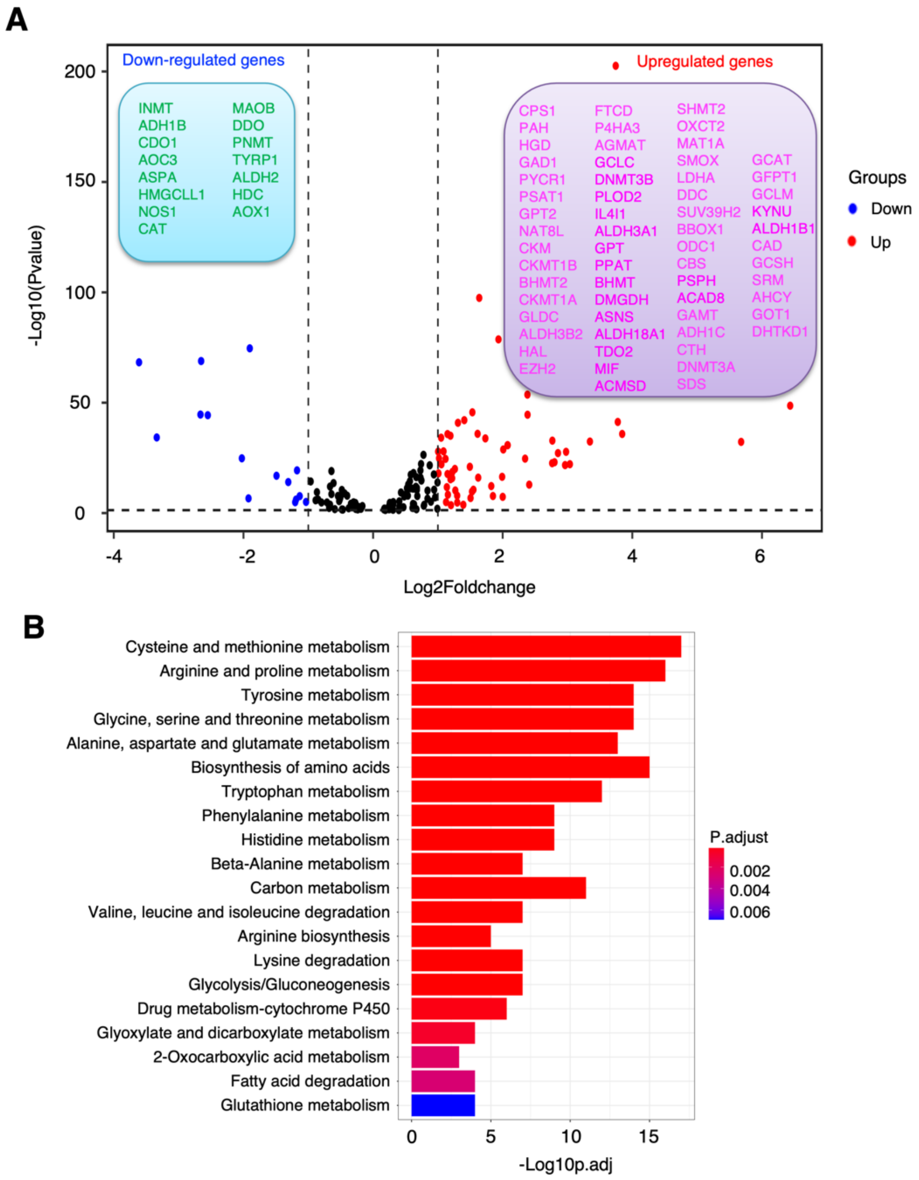

Supplementary Figure 2. Differential analysis expressions of tumor-specific amino acid-related genes. (A) A volcano plot showing the distribution of differentially expressed genes related to amino acid metabolism pathways between the tumor and adjacent non-neoplastic samples. The TAAMGs were labeled in the plot using gene names. Red dots: upregulated genes; blue dots: downregulated genes. (B) The bar plot shows the pathway enrichment analysis results of TAAMGs using KEGG datasets.

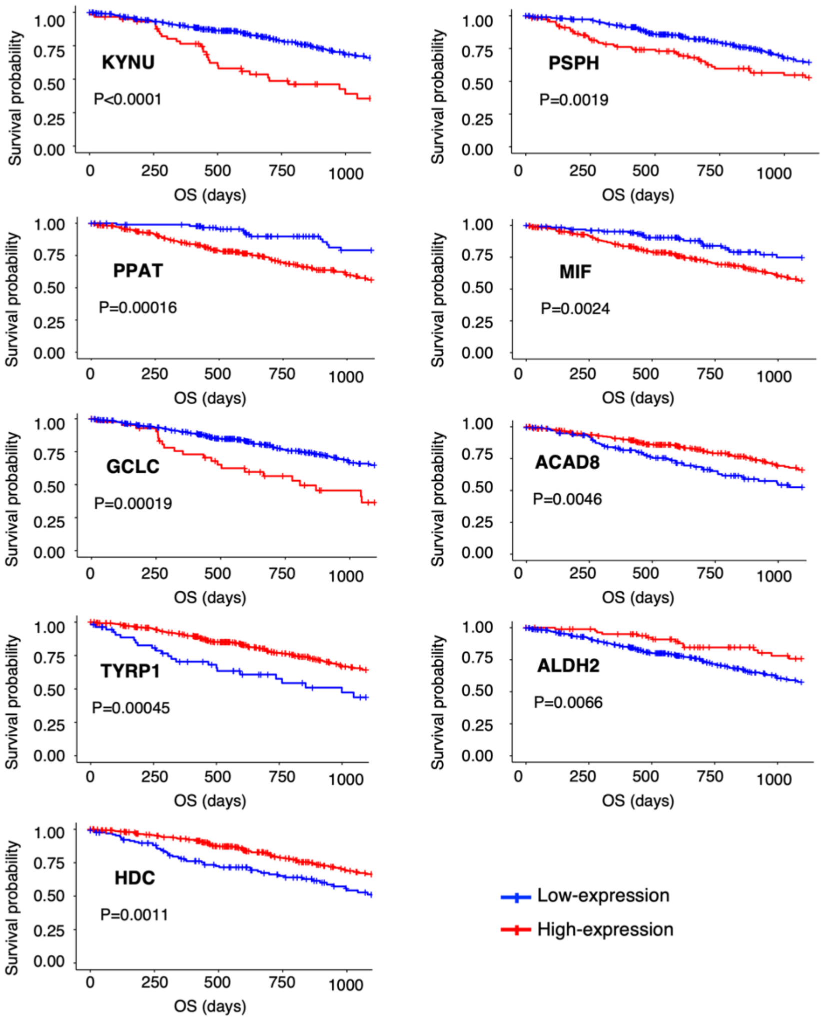

Supplementary Figure 3. Survival analysis for the nine key PTAAMGs. KM survival curve stratified by gene expression levels of the nine key PTAAMGs in the high- and low-expression groups in the TCGA cohort. Patients were divided into low- and high-expression groups based on the optimal AUC cutoff. *P*: log-rank test.

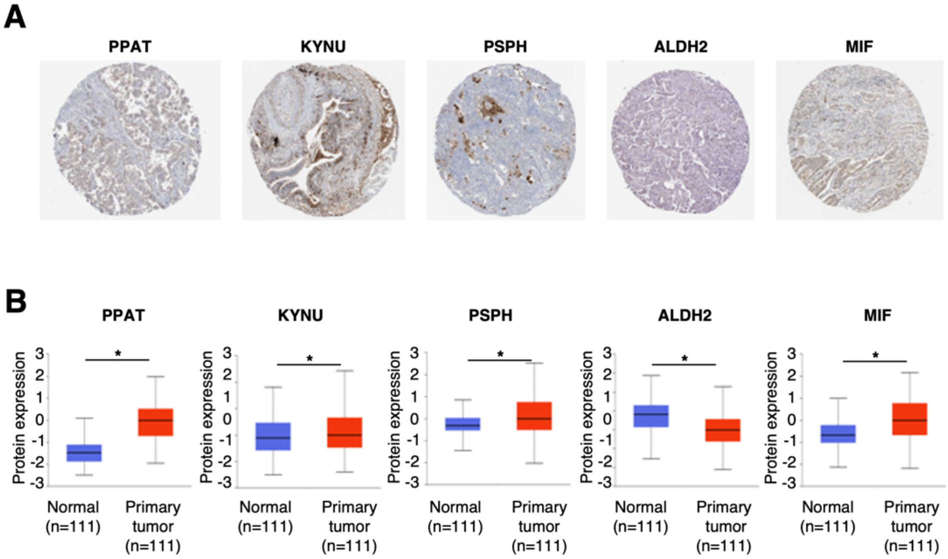

Supplementary Figure 4. Protein expression of the key TAAMGs in TCGA cohort. (A) Immunohistochemical staining revealed the protein expression levels of PPAT, KYNU, PSPH, ALDH2, and MIF in tumors and adjacent non-neoplastic tissues in LUAD from the HPA dataset. (B) Quantitative validation of PPAT, KYNU, PSPH, ALDH2, and MIF protein expression in 111 pairs of tumors and corresponding adjacent non-neoplastic tissues from CPTAC mass analysis (paired *t*-test, $^*P < 0.05$).

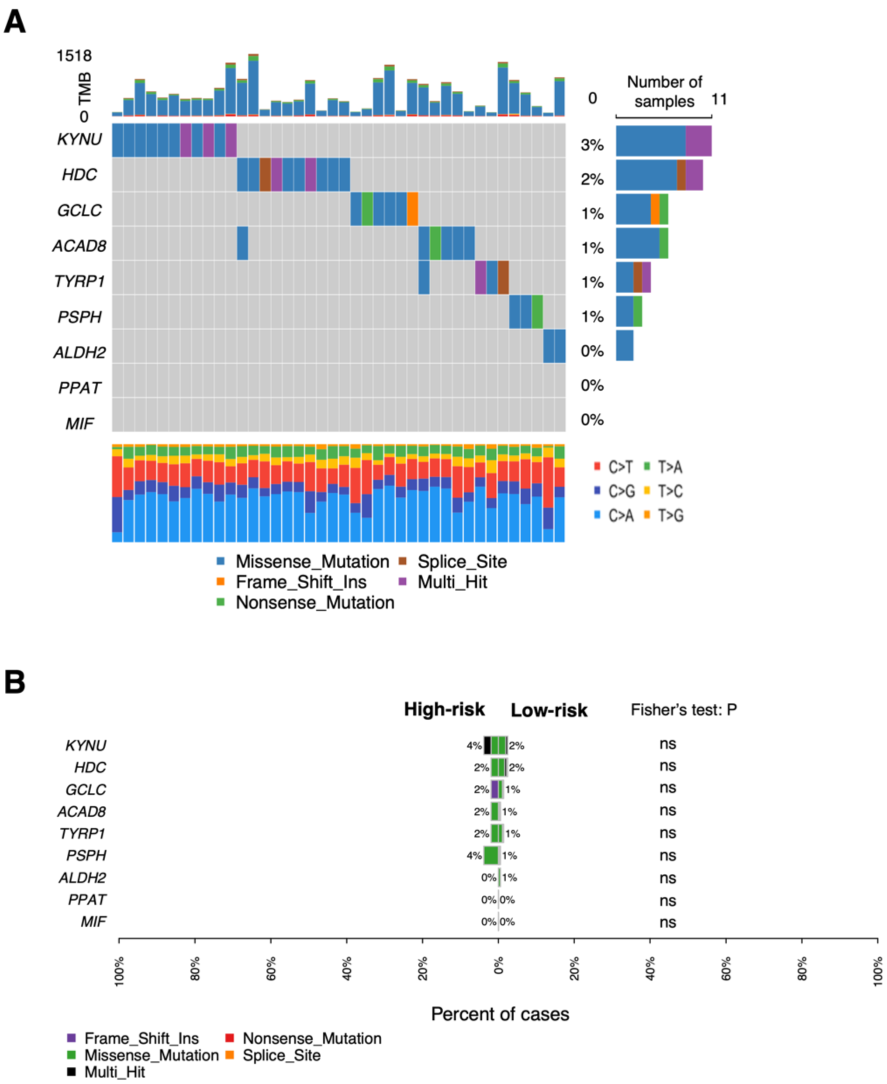

Supplementary Figure 5. Somatic gene mutation profiles of the key TAAMGs in TCGA cohort. (A) Waterfall diagram showing the mutation status of nine key TAAMGs. (B) The mutation frequencies of the nine key TAAMGs were compared between the high- and low-risk groups. *P*-values using Fisher's exact test; ns: not significant.

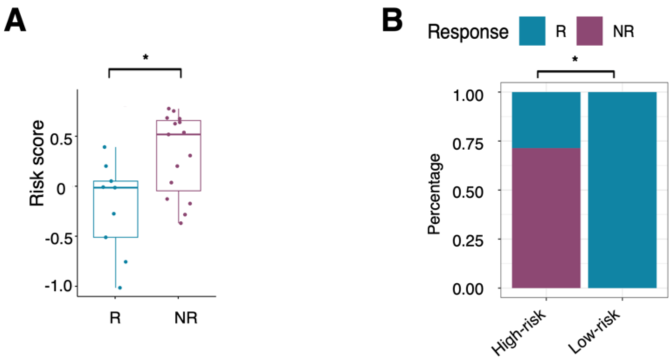

Supplementary Figure 6. Investigation of the prediction ability of the PTAAMPG-Sig on response to chemotherapy and ICI combine therapy in the GSE207422 cohort. (A) Boxplot showing the distribution of the PTAAMG-Sig risk scores in patients with different immunotherapeutic responses in GSE207422. R: Responders; NR: Non-responders. Wilcoxon rank sum test, **P* < 0.05. (B) The proportion distribution of patients with immunotherapeutic responses in the PTAAMG-Sig high- and low-risk groups in GSE207422. R: Responders; NR: Non-responders. Chi-squared test, **P* < 0.05.

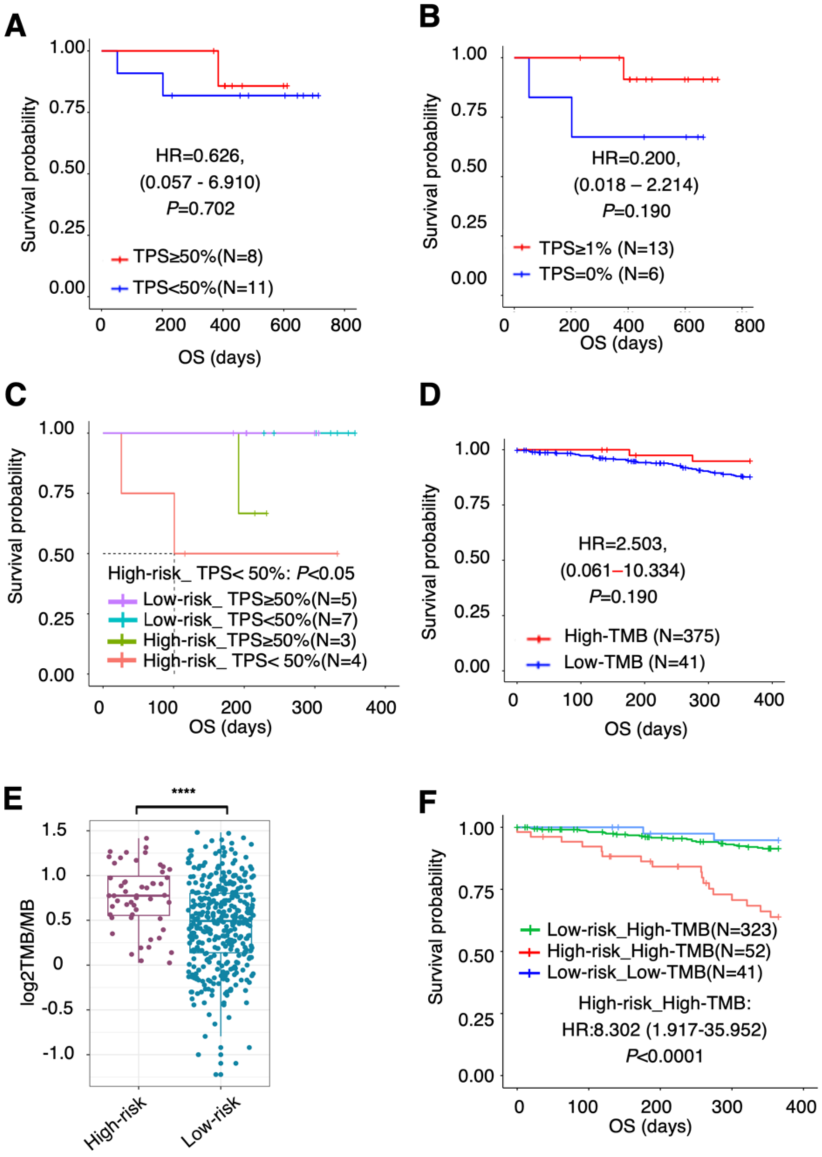

Supplementary Figure 7. Correction between the PTAAMA-Sig and PDL1 expressions or TMB levels in OS prediction outcome. (A) KM curve analysis for OS in the PD-L1 expression levels with tumor proportion score (TPS) high (≥50%) or low (<50%) subgroups. *P*-value: log-rank test. (B) KM analysis for OS in the subgroups stratified by the positive (≥1%) or negative (=0%) PD-L1 TPS. *P*-value: log-rank test. (C) KM survival curve of OS is stratified by the combination of the PTAAMG-Sig scores and PD-L1 TPS, including Low-risk_TPS ≥ 50% as the control, Low-risk_ TPS < 50%, High-risk_ TPS ≥ 50%, High-risk_ TPS ≥ 50% four groups. *P*-value: log-rank test. (D) KM survival curve of OS was stratified by the log2 (TMB/MB) levels in the TCGA cohort. Patients were divided into Low- and High-TMB groups at a cutoff value of -0.167, based on the optimal AUC cutoff. *P*-value: log-rank test. (E) Boxplot showing the distribution of log2TMB/MB in the subgroups stratified by the PTAAMA-Sig in the TCGA cohort. Wilcoxon rank sum test, ****$P < 0.0001$. (F) KM survival curve of OS is stratified by the combination of the PTAAMG-Sig scores and TMB levels, including Low-risk_Low-TMB as the control, Low-risk_High-TMB, and High-risk_High-TMB three groups.

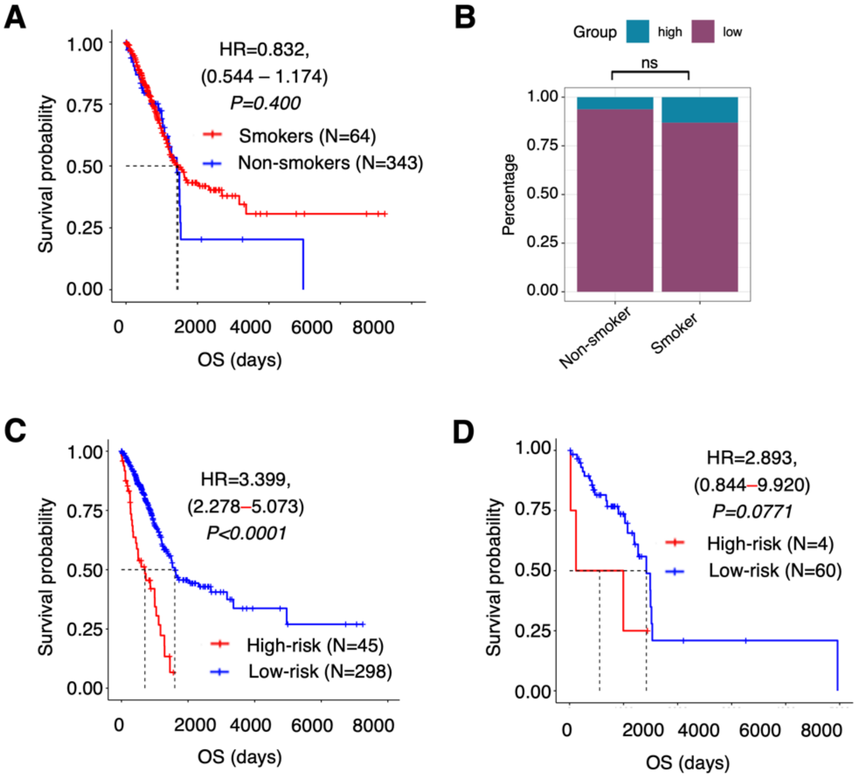

Supplementary Figure 8. Efficacy of the PTAAMG-Sig in OS predicting risk in smokers and non-smokers. (A) KM survival curve of OS was stratified by smokers (less than 100 cigarettes smoked in a lifetime) and non-smokers (includes current smokers and current reformed smokers) in TCGA-LUAD cohort, *P*-value: log-rank test. (B) The proportion distribution of the PTAAMG-Sig high- and low-risk with smoking status with non-smokers and smokers. low: Low-risk; high: High-risk. Chi-squared test, ns: not significant. (C)KM survival curve of OS was stratified by the PTAAMG-Sig high- and low-risk in smokers. Smokers were divided into low- and high-risk groups, *P*-value: log-rank test. (D) KM survival curve of OS was stratified by the PTAAMG-Sig high- and low-risk in non-smokers. *P*-value: log-rank test.

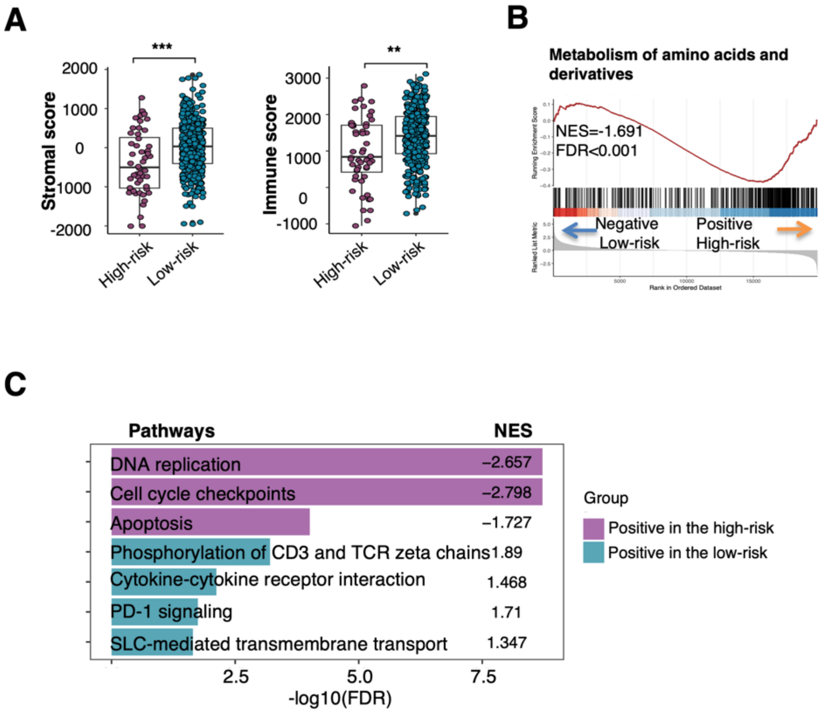

Supplementary Figure 9. Differentially expressed genes in tumor microenvironment between the PTAAMG-Sig high- and low-risk groups in TCGA cohort. (A) Boxplots depicting the stromal and immune scores using the ESTIMATE algorithm in the PTAAMG-Sig low- and high-risk groups. Wilcoxon rank sum test, $^{**}P < 0.01$; $^{***}P < 0.001$. (B) GSEA enrichment plots of differentially expressed genes between the PTAAMG-Sig high- and low-risk groups in the "Metabolism of amino acids and derivatives" GO term. (C) A bar plot of differentially expressed genes between the high- and low-risk PTAAMG-Sig groups in pathways from the Reactome and KEGG databases. Purple: significant positive enrichment in the high-risk group; blue: significant positive enrichment in the low-risk group.

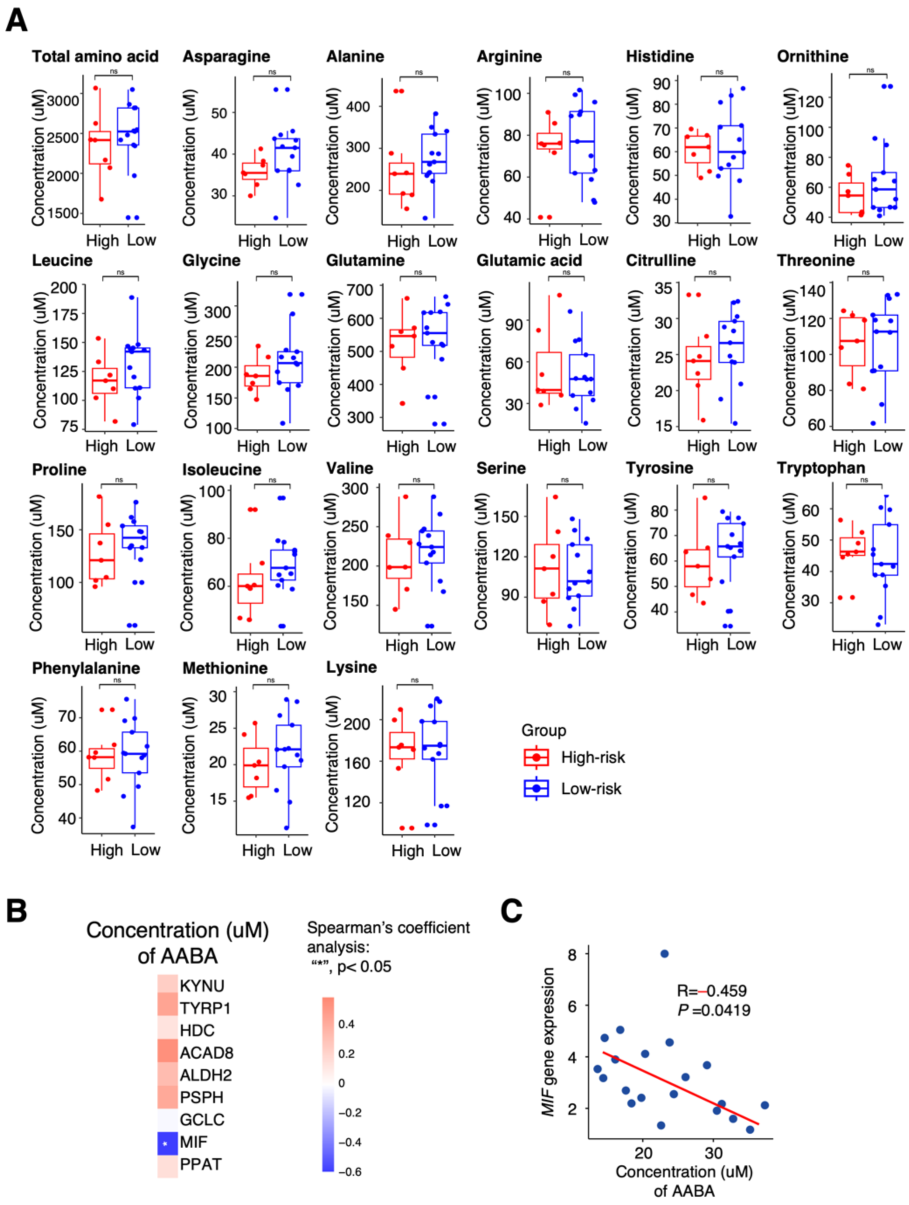

Supplementary Figure 10. Differential distribution of the concentrations of PFAAs in blood according to the PTAAMG-Sig risk scores. (A) Boxplots of the concentration differences of the other 20 PFAAs and total amino acids in the blood between the low- and high-risk groups. Wilcoxon rank sum test; ns: not significant. (B) Heatmap of the correlations between the concentration of AABA and gene expression levels of the key PTAAMGs in tumor tissues. Spearman's coefficient analysis, *$P < 0.05$. Blue: negative correlation; Red: positive correlation. (C) Scatter plots presenting the correlations between the concentration of AABA and *MIF* gene expression levels in tumor tissues. Spearman's correlation analysis.
